# Relation between Shear Stresses and Flexural Tensile Stresses from Standardized Tests of Extracted Prismatic Specimens of an SFRC Bridge Girder

**DOI:** 10.3390/ma15238286

**Published:** 2022-11-22

**Authors:** Alfredo Quiroga Flores, Rodolfo Giacomim Mendes de Andrade, Michèle Schubert Pfeil, Joaquim A. O. Barros, Ronaldo Carvalho Battista, Olga Maria Oliveira de Araújo, Ricardo Tadeu Lopes, Romildo Dias Toledo Filho

**Affiliations:** 1 Civil Engineering Department, COPPE, Universidade Federal do Rio de Janeiro, Rio de Janeiro 21941-972, Brazil; 2 Buildings and Civil Engineering Department, Instituto Federal do Espírito Santo, Vitória 29040-780, Brazil; 3 ISISE, Department of Civil Engineering, School of Engineering, University of Minho, 4800-058 Guimarães, Portugal; 4 Nuclear Engineering Program, COPPE, Universidade Federal do Rio de Janeiro, Rio de Janeiro 21941-972, Brazil

**Keywords:** shear behavior, steel fiber-reinforced concrete, box girder, fiber distribution

## Abstract

Experimental research on the direct shear behavior of fiber-reinforced concrete is often carried out using prisms molded with specific dimensions for a standardized test. However, the flow of fresh concrete in these molds can be different than in the case of a full-scale structural element. This is important considering that the flow direction highly influences the distribution and orientation of fibers. In addition, most of the studies did not relate their shear results to other mechanical properties. In contrast, this study attempted to deepen the experimental knowledge of the crack propagation of a steel fiber-reinforced concrete (SFRC) used in a full-scale prototype of a bridge box girder built in the laboratory. Prismatic specimens were sawn from webs and top flanges of this prototype. Serving as references, additional specimens were molded in wooden boxes. In a previous study of our research group, both had been tested under a three-point notched bending configuration maintaining test conditions proportional to the EN14651 specifications. From each of the previously flexurally tested specimens, two prismatic specimens suitable for the Fédération Internationale de la Précontrainte (FIP) shear test setup were extracted by adopting a cutting methodology that avoided the damage induced by the flexural tests to be part of the FIP specimens. These FIP specimens were tested in almost pure shear loading conditions for assessing the performance of SFRC. Computer tomography images and photos of the shear failure faces were used to determine the distribution and density of fibers. The results demonstrated that the peak loads were proportional to the fiber density at the shear failure section. Assuming that the SFRC conditions of the webs were representative of a common batching procedure in the construction industry, the results from the tests in specimens extracted from these webs were adopted to establish shear stress/flexural tensile stress ratios vs. crack mouth opening displacement curves. The curves belonging to cross-sections of a similar fiber density in the shear and flexural cases allowed for the proposal of a normalized crack-dilatancy relation composed of three stages of the crack propagation. In addition, a trilinear crack width–slip relation was established using the same set of specimens. The relevancy of this proposal is that the shear response can be estimated from a widely accepted standardized flexural test, which demands a simpler instrumentation and is also easier to execute than the shear setup.

## 1. Introduction

The load carrying capacity at serviceability and at the ultimate limit state conditions of constructive systems made by plain and reinforced concrete (PC and RC, respectively) increases with the contribution of fiber reinforcement due to the ability of fibers bridging cracks to support the load transferred between the crack faces and to restrain their width and sliding. Therefore, the inclusion of fibers results in a matrix of higher energy absorption capacity and post-cracking strength than the PC, but the fiber reinforcement efficiency depends on the volume and type of fibers, the quality of the matrix and the batching methods involved due to the influence of fiber distribution and orientation [1,2,3,4,5,6,7]. These advantages have been demonstrated in the application of various structural elements, such as slender beams [8,9,10], deep beams [11,12,13], slabs [14,15,16], columns [17] and tunnel lining segments [18,19].

When fibers work as the shear reinforcement of beams, they are an efficient substitute of conventional stirrups by providing a diffuse reinforcement, delaying the failure mechanism and making it feasible to change the failure from brittle to ductile [20]. When the shear loads cause cracking, the resisting mechanisms are ensured by the concrete matrix, aggregate interlock, fiber pull-out and dowel effect of flexural tensile reinforcement (structural elements failing in shear always have conventional flexural reinforcement) [21]. During the cracking process, normal and shear stresses are transferred from one side to the opposite side of the crack, while two kinematic phenomena occur: crack opening, also known as crack width, and the sliding of the contact areas, also known as slip or overriding [22]. Throughout this process, the matrix suffers from permanent deformation due to high contact stresses. On the condition that the strength of the cement paste of the matrix is lower than the aggregate resistance, as occurs in a conventional-strength concrete, the crack path can propagate around the aggregates. Conversely, the crack path can cross through the aggregates in high strength. On the other hand, the aggregate interlock is considered the main support factor of the shear transfer mechanism. It was found that when the crack width exceeds half the maximum dimension of aggregates, the aggregate interlock resisting mechanism decreases pronouncedly [22]. Moreover, the fibers play a subordinate role by limiting both the crack opening and sliding, which ends when they are fully pulled out or ruptured [21,23]. The flexural and shear stiffness of the fibers are disregarded due to their relatively small cross-section [23].

The distribution and orientation of fibers are highly influenced by the flow conditions of fresh concrete; fibers have the tendency to align orthogonally to the flow direction [24]. Hence, their contribution to the shear mechanical strength varies depending on the number of fibers crossing a determinate cross-section and the angle they form with an orthogonal axis to the cracked surface [25]. Khanlou et al. [26] experimentally verified that the maximum shear capacity increases with the concrete strength and, mainly, with the fiber volume. In addition, empirical and semiempirical equations based on compressive strength and fiber volume were proposed to calculate the shear strength of a fiber-reinforced concrete (FRC) [26,27,28,29,30]. However, the validity of these equations is mostly restricted to the database from which they were calibrated. Subsequently, theoretical models were also proposed [21,23,30], combining the formulations that simulate the aggregate interlock resisting mechanisms [22,31,32] with those modeling the fiber pull-out resisting mechanisms and fiber orientation [33,34,35].

Various experimental setups have been employed to determine the shear behavior of PC, RC and FRC; however, the difficulty of achieving pure shear in the cracking plane is a common characteristic. Overall, all these tests are more demanding in instrumentation in comparison with compression and bending cases, which are common for material characterization. Three setups were found to be the most utilized: the Z-type push-off test initially conceived for PC and RC [36], the modified JSCE-G 553 Japanese test [37] and the FIP four-point load test [38]. Tests for assessing the influence of rebars crossing the failure plane on the shear resisting mechanics due to the arrestment of the relative movement of crack faces have been carried out [39,40,41], where the confinement provided by stresses applied in the direction orthogonal to the crack plane was also considered [30]. In some studies, specimens were previously cracked [42] to assess the influence of a certain relative pre-movement of the faces of the shear failure crack on the shear resisting mechanisms. 

Most of the research campaigns utilized specimens molded with specific dimensions for the experimental tests. Considering that the mechanical response of FRC is highly influenced by the pouring method, among other variables, the execution of experimental tests with specimens extracted from a structure that can be more representative of the real behavior than molded specific-dimension prisms will provide more reliable results in terms of extracting conclusions and their use for supporting design guidelines and analytical and numerical models. Furthermore, few direct-shear studies provided a comprehensive characterization of the material. In addition to the shear results, most of them provided only the compressive strength. Therefore, the relationships between shear behavior and other mechanical properties have not been explored. In this context, it is desirable to relate the shear performance to results from an extensively adopted test, such as the EN 14651 flexural experiment [43]. The EN14651 standard determines the flexural response of an FRC, with emphasis on the cracked stage. After crack initiation, the post-cracking flexural-tensile stress and crack width are obtained in the notched cross-section. Within this frame of reference, this study seeks to attend these two research interests. First, post-cracking properties are determined in prismatic specimens sawn from an R/SFRC full-scale box girder prototype intended for bridge purposes [44]. Second, an effort was made to establish correlations between shear and flexural tensile stresses and the crack mouth opening displacement with the final objective of proposing a normalized crack–dilatancy relation. By combining this information with a crack width–slip relation, this proposal allows shear capacity to be estimated from the results of a standardized flexural test. A box girder for precast bridge purposes was chosen because there is an interest in taking advantage of the gain of post cracking strength and the durability of SFRC in a structural element used in the worldwide infrastructure system. Concrete bridges require periodic maintenance and repair. SFRC properties can increase both the period between maintenance activities and the structure lifespan. However, the efficiency of the mechanical contribution of fibers is highly influenced by the concrete production methods; thus, the construction of a laboratory prototype model of a stretch of the bridge is highly relevant after the design. The extraction of specimens from this prototype allows for the prediction of the fiber dispersion and mechanical performance before the final construction of the bridge.

## 2. Materials and Methods

### 2.1. The R/SFRC Box Girder Prototype

**Figure 1:**
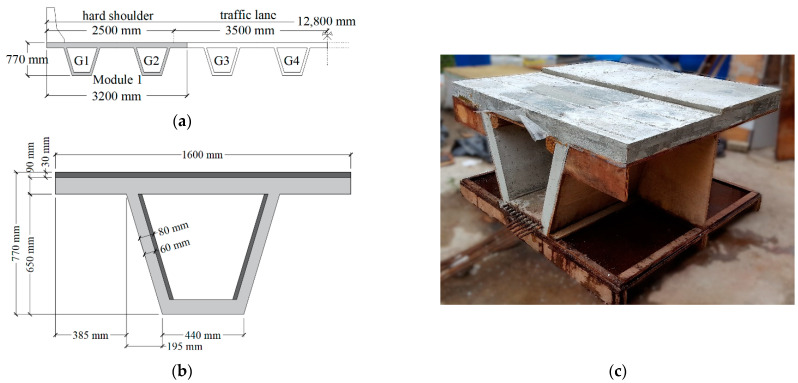
Full-scale prototype of a box girder of an R/SFRC bridge [44]: (**a**) bridge cross-section formed by modules composed of two box girders; (**b**) dimensions of the cross-section of a box girder; (**c**) the box girder prototype during the removal of formwork.

In order to understand the origin of the prisms experimentally tested, a description of the full-scale box girder prototype is given. In a previous study [44], an R/SFRC bridge was designed to support a Class 1-B Motorway [45,46]. The bridge width was 12.80 m, projected to attend two 2.5 m hard shoulders and two 3.5 m traffic lanes, as shown in Figure 1a. The cross-section is composed of four precast modules which are formed by two box girders whose dimensions are presented in Figure 1b. Then, a full-scale prototype of half a module (i.e., a box girder) was built in the laboratory. This prototype had two dimensions for both the web thickness and top flange height. The half-length of the prototype had a web thickness of 6 cm to represent the cross-section in the central span of the bridge, and the other half had 8 cm to stand for the cross-section near the supports. Additionally, the half-length of the mock-up had 9 cm top flanges to represent the module during construction in a precast factory and transportation stages. Then, a 3 cm layer was added to the other half to depict the final dimension after all the modules were placed together to form the bridge in the construction site. Moreover, the design was based on Model Code 2010 [47], utilizing the material properties of a concrete dosage of the COPPE research institute [48]. In Figure 1c, the box girder prototype that is 1.5 m in length during the removal of the forms is presented. Although detailed characteristics of the wooden forms, rebar disposition, batching steps, curing and demolding can be found elsewhere [44], the fundamental information for a comprehensive understanding of this study is provided in this section.

**Figure 2:**
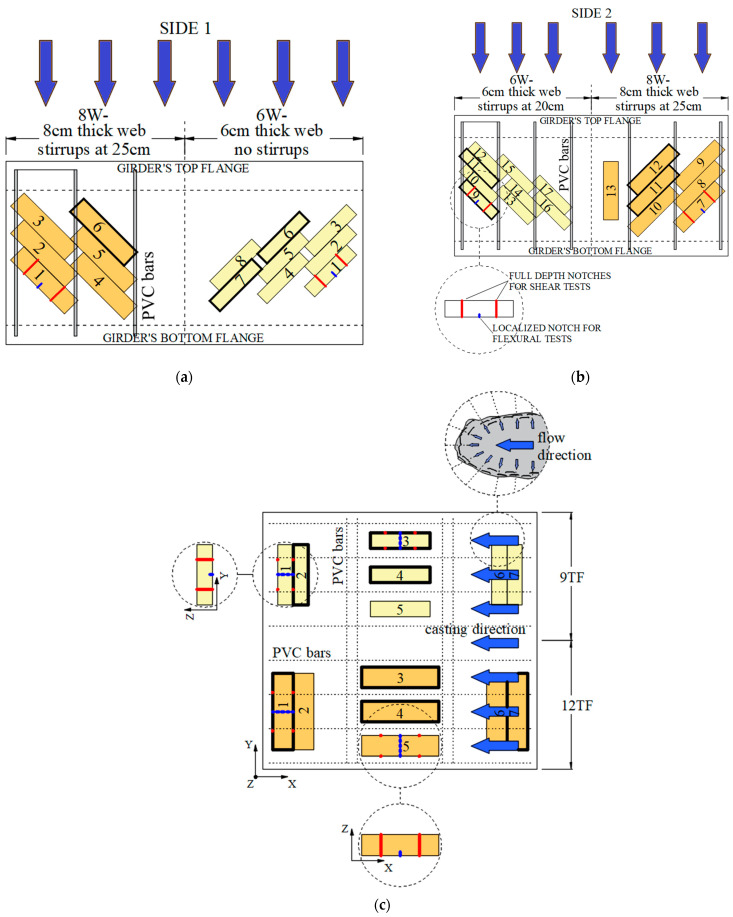
Identification of extracted prisms from: (**a**) mock-up’s web’s Side 1; (**b**) web’s Side 2 and (**c**) top flanges.

Given that this prototype was built to determine the flexural and shear response of extracted specimens from different locations of its structure, steel bar reinforcement was replaced by 15 mm PVC tubes (Figure 2a–c) to facilitate the sawing of prisms. In Figure 2a,b, the flow direction during the pouring of fresh SFRC in the web formwork is indicated by the blue arrows, from the top to the bottom direction. On the other hand, the placement of fresh concrete in the top flanges is detailed in Figure 2c, where the *x* and *y* axes represent the transverse and longitudinal directions of the mock-up, respectively. The placement of concrete started by pouring the content of the first buckets of fresh SFRC along the right corner. The flow direction of the content of every bucket is described in the top right corner of Figure 2c. The fresh concrete of the subsequent buckets was poured on the left side of the previous ones; hence, the process was repeated until the top flange forms were fulfilled. When the concrete was hardened, the prisms were marked and numbered, as shown in Figure 2a–c. Later, 6 × 6 × 25 cm^3^ (6W) and 8 × 8 × 32 cm^3^ (8W) prisms with a 45-degree inclination were extracted from the webs (Figure 2a,b). Similarly, 9 × 9 × 35 cm^3^ (9TF) and 12 × 12 × 45 cm^3^ (12TF) prisms were obtained from the top flanges (Figure 2c). In a previous study [44], the prisms were tested under a three-point notched bending setup following the procedure and proportional dimensions of the EN14651 standard [43]. The location of the notches for flexure is depicted with a blue line in the prisms in Figure 2a–c. In the present study, the prismatic specimens marked with a thick black line in Figure 2a–c were employed for shear tests. Analogously, the reference specimens, batched in prismatic molds and having the same size as the extracted prisms, were tested under flexure and were later shear tested.

### 2.2. Materials and Mix Proportions

**Figure 3:**
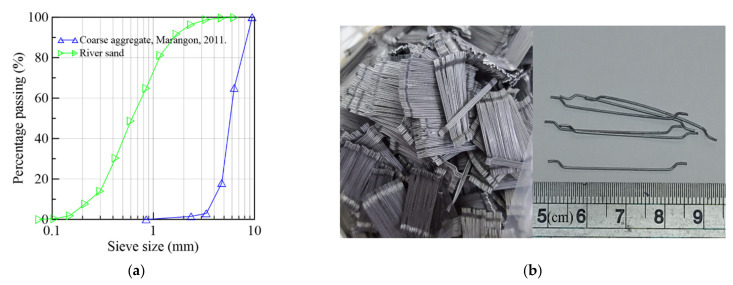
(**a**) Size distribution of aggregates [48] and (**b**) fibers.

An SFRC with self-compacting properties based on a preceding concrete [48] developed at the COPPE institute was devised for the prototype. Steel fibers with an aspect ratio of 65, a 35 mm length and a 0.55 mm diameter, representing 1.5% volume of the mixture, were employed. The composing materials are detailed in Table 1. The granulometry of the coarse aggregate and river sand is presented in Figure 3a, and the fibers used are presented in Figure 3b. In the fresh state, the matrix presented 605–645 mm slump-flow and around 51 s in the V-funnel test.

### 2.3. Mechanical Tests

**Figure 4:**
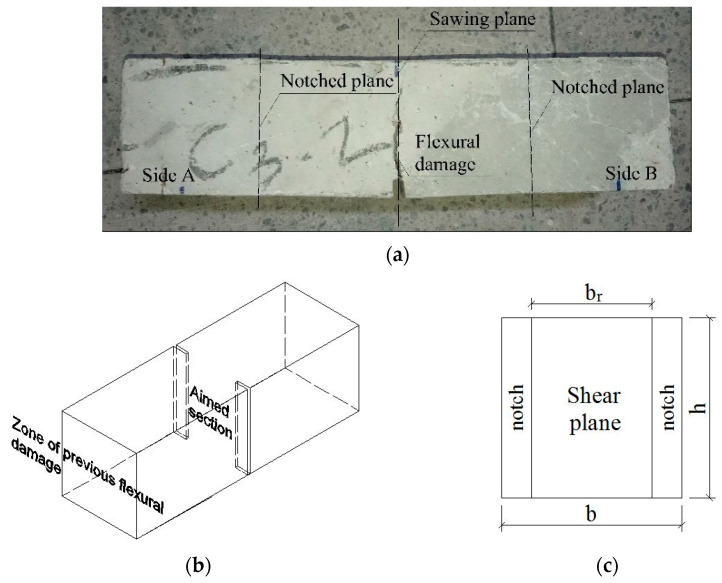
Preparation of the prisms for the FIP shear test: (**a**) sawing plane and notching of a prism after the flexural test; (**b**) double-notched prism for the shear test; (**c**) cross-section of the sliding plane.

Shear tests were carried out based on the standardized FIP test [38]. However, it was necessary to prepare the prisms first because they had been flexurally tested. Thus, the reference and mock-up prisms were sawn where flexural cracking had occurred, namely, following the cutting plane depicted in Figure 4a. The two remaining parts, Side A and Side B, were then notched in both lateral surfaces, following the notched planes described in Figure 4a, to promote shear failure localization in the aimed section (Figure 4b). The position of the prisms with respect to the mock-up is shown in Figure 2a–c along with their notches for shear tests, represented with red lines. Furthermore, the full (b·h) and reduced ($b_{r}\cdot h)$ cross-sections are defined in Table 2 and shown in Figure 4c. The notch width was equal to the space left on the prism by the cutting disc, which was 2.5 mm, on average.

**Figure 5:**
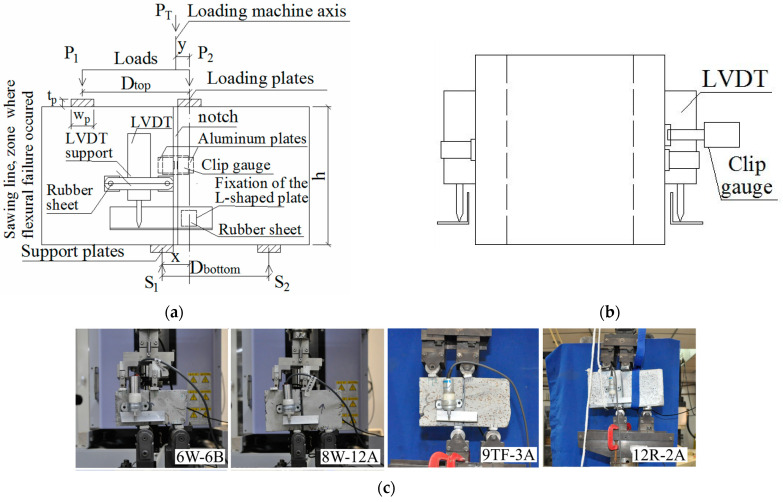
Setup based on the FIP test [38]: (**a**) front side of the test setup (the rear side has an equal setup with the addition of a clip gauge, as shown in the dashed line); (**b**) side view of the test setup; (**c**) tests at different loading stages.

Finally, the shear test was mounted, consisting of the application of four concentrated loads, as detailed in Figure 5a–c. Both the acting (*P*_1_ and *P*_2_) and support (*S*_1_ and *S*_2_) loads were separated by spans D ($D=D_{top}=D_{bottom}$) (see Table 2); for specimens of the 12 × 12 cm^2^ full cross-section, $D_{top}$ is different from $D_{bottom}$ due to a limitation of the spacing between the top loads in the steel setup available in the laboratory. This modification of the FIP test did not alter the null moment in the notched cross-section since the total acting load, $P_{T}$ (Equation (1)), was positioned between *P*_1_ and *P*_2_ to satisfy this requirement. To do so, the $S_{1}$ and $S_{2}$ reaction forces were obtained from the force equilibrium (Equations (2) and (3)), where *x* represents the separation between the *S*_1_ and *P*_2_ loads and *y* represents the distance between the $P_{T}$ and *P*_2_ loads (Figure 5a). Then, the position of the total acting load (*y* in Figure 5a) to guarantee a null moment in the notched cross-section was determined from Equations (4) and (5). An additional verification was carried out: the requirement for $S_{2}$ to maintain its direction was determined, as depicted in Figure 5a, namely, to avoid the prism lifts from the $S_{2}$ support after the reposition of the $P_{T}$ load. This is satisfied, as defined by Equation (6), which is based on Equation (3), i.e., it is determined that the y distance cannot be higher than the x distance. Therefore, Equations (5) and (6) ensured the proper performance of the shear test. The shear stress in the notched plane was calculated according to Equation (7) for the dimensions of every prism size defined in Table 2.

A linear variable displacement transducer (LVDT) was fixed on both the front and rear surfaces of the prism to measure the slip of the sliding plane (Figure 5a). A clip gauge was put in the notch of the rear side to measure the crack width, as drawn in a dashed line in Figure 5a and also depicted in the side view (Figure 5b). The tests were executed under displacement control at a rate of 0.1 mm/min, consistent with the values found elsewhere [49], by using both aforementioned LVDTs to measure the vertical deformation of the prims. To avoid stress concentration, bearing plates of width ($w_{p}$) × thickness ($t_{p}$) × length ($l_{p}$) dimensions were used, where $w_{p}$ and $t_{p}$ are the cross-section dimensions of every plate described in the bearing plate below $P_{1}$ in Figure 5a, and $l_{p}$ is equal to the *b* dimension from the full cross-section. Then, 15 × 5 × $l_{p}$ mm^3^ was used for the 6 and 8 cm specimens. For the 9 cm and 12 cm prisms, 20 × 20 × $l_{p}$ mm^3^ and 25 × 10 × $l_{p}$ mm^3^ load plates were employed, respectively. Figure 5c presents the specimens randomly chosen at different loading stages, representing the four sizes tested. Due to the small dimensions of the specimens, no additional LVDTs could be placed horizontally to measure a possible rotation of the cracking plane; this was estimated by taking photos every twenty to thirty seconds. The label to identify every specimen is indicated in Table 2. The first number means the size of the square cross-section (6, 8, 9 and 12 cm). The following letter indicates the part of the mock-up from which the specimen originates (W = web, TF = top flanges, R = reference, molded). The X represents the specimen number in Figure 2a–c, and Y can be A or B, i.e., the side of the specimen sawn in two halves (Figure 4a).

(1) $$P_{T}=P_{1}+P_{2}$$

(2) $$S_{1}=P_{T}\left(1-\left(\frac{x-y}{D_{bottom}}\right)\right)$$

(3) $$S_{2}=\frac{P_{T}}{D_{bottom}}\left(x-y\right)$$

(4) $$\sum M_{notched\;section}=P_{1}\left(D_{top}-\frac{x}{2}\right)-S_{1}\frac{x}{2}=0$$

(5) $$y=\frac{D_{top}\left(xD_{bottom}-x^{2}\right)}{2D_{top}D_{bottom}-xD_{bottom}-xD_{top}}$$

(6) $$S_{2}>0\;\mathrm{if}\;x-y>0\to x>y$$

(7) $$\tau =\frac{P_{1}-S_{1}}{b_{r}h}$$

### 2.4. Edge Effects

There are two edge factors, due to the sawing and wall, that are applied on the processed data of the mechanical response of the specimens. When sawing occurs during the extraction of specimens from the mock-up, the fibers near the sawn side are also cut; thus, it is considered that half of the fibers are not anchored anymore over a width of the half fiber length $\left(l_{f}/2\right)$ along the sawn surface. According to the Association Française de Génie Civil (AFGC) [50], a sawing factor ($f_{saw})$ of 0.5 is recommended to be applied on this zone. The second edge factor is related to the effect of the formwork acting like a wall on the fiber orientation. In a 3D fiber orientation, fibers can rotate freely in any direction; however, a formwork modifies this orientation, causing a 2D alignment of fibers in the close concrete zone of $l_{f}/2$ width. Similarly, in the corners where two adjacent forms meet, a quasi 1D orientation occurs. The corresponding orientation factors proposed by Krenchel [51] are $\eta_{3D}=$ 0.5, $\eta_{2D}=2/\pi =$ 0.637 and $\eta_{1D}=$ 0.84. The AFGC recommends the use of average orientation factors: $\eta_{3D}=$ 0.41, $\eta_{2D}=$ 0.597 and $\eta_{1D}=$ 0.841, which were used in the present study. In the literature, there are other orientation factors [52,53] that vary slightly in comparison to the aforementioned ones. The AFGC recommends considering these edge effects in order to ensure that the results are representative of a 3D case; then, the shear cross-section is split into sub-areas depending on the factors that apply. In other words, a unique factor, or a combination of sawing and fiber orientation factors, can occur in a determined sub-area.

**Figure 6:**
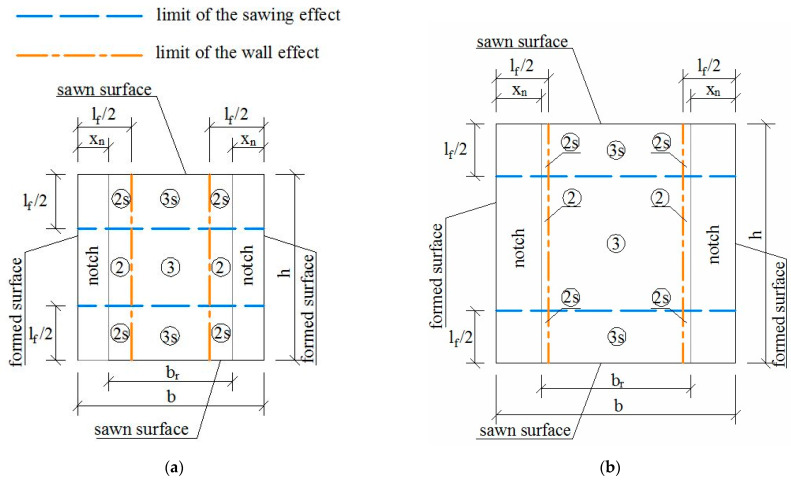

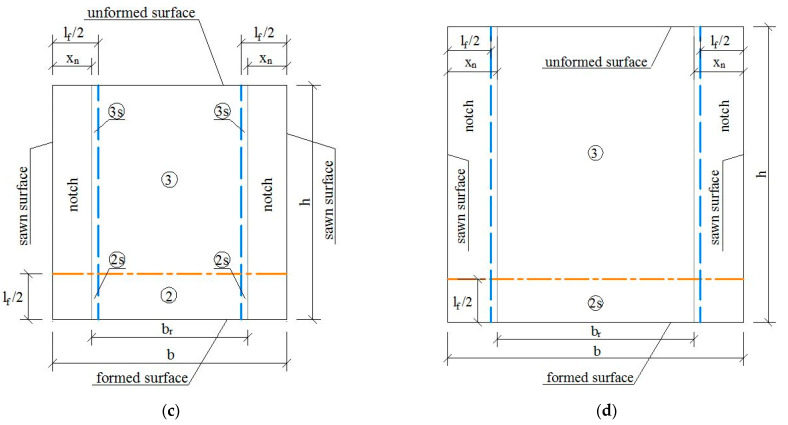
Edge effects of the sawing and wall on the cross-section areas of the prisms from the mock-up: (**a**) 6W, (**b**) 8W, (**c**) 9TF and (**d**) 12TF.

**Figure 7:**
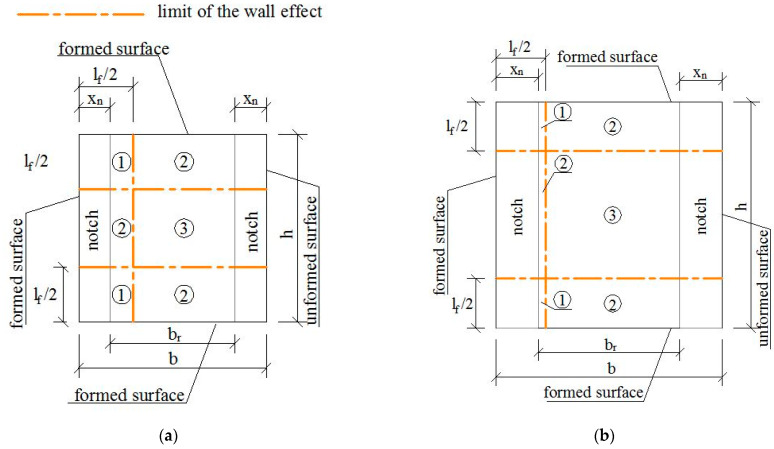

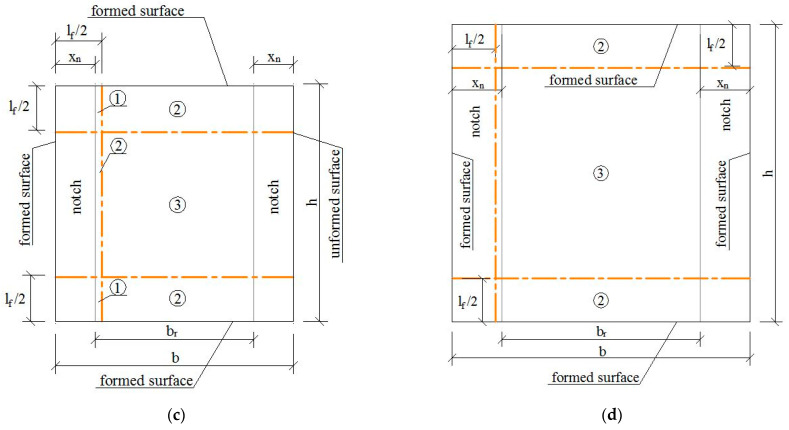
Edge effects of the sawing and wall on the cross-section areas of the reference prisms: (**a**) 6R, (**b**) 8R, (**c**) 9R and (**d**) 12R.

Thus, for any shear cross-section of any prism of the prototype, its position in Figure 2 was considered, and sub-areas were defined depending on the edge factors affecting each of them, as shown in Figure 6. The notch depth is represented by $x_{n}$. The shear cross-section area is delimited by $b_{r}$ and *h*. The limit of the wall and sawing effects is defined by two different lines, as depicted at the top of Figure 6. The edge effects acting on any sub-area are described in Figure 6 by using a number (1, 2 and 3 for 1D, 2D and 3D fiber orientations, respectively) when the wall effect is the unique effect, or by a number plus the s letter (1 s, 2 s and 3 s) when a combination of wall and sawing effects acts. For example, in Figure 6a, the sub-area in the top-left corner (2 s) is affected by a wall factor of the 2D fiber orientation and the sawing effect. Subsequently, an average efficiency factor ($f_{avg})$ of the shear cross-section is determined with Equation (8), where $A_{s,i}$ is the *i*th region affected by the saw cut, $\eta_{j,i}$ is its fiber orientation factor (*j* = 1, 2 or 3 for 1D, 2D or 3D), nsr is the number of regions affected by the saw cut, $A_{k}$ is a *k*th region not affected for the saw cut, $\eta_{j,k}$ is its orientation factor (*j* = 1, 2 or 3 for 1D, 2D or 3D) and m is the number of regions not affected by the saw cut. Their effects proportionally influence the size of the sub-areas on which they act. The edge factor ($f_{edge}$) is determined as a relative value with respect to a 3D random orientation factor in Equation (9), and, finally, the shear stress registered during the test ($\tau_{raw}=\left(P_{1}-S_{1}\right)/\left(b_{r}\cdot h\right)$) is corrected by the edge factor, as defined in Equation (10). Following the same criteria, the shear response of any reference prism that was cast in a wooden mold was also affected. The division in sub-areas for every prism size of the reference set is presented in Figure 7. The edge effects had already been considered in the flexural tensile stresses in a previous study [44]. The flexural tensile stress (σ) was calculated as $\sigma =\frac{f_{R,j}}{f_{edge}}$, where $f_{edge}$ corresponds to the edge effects due to the sawing and formwork and $f_{R,j}$ is the flexural tensile stress defined by the EN 14651 standard, calculated as $f_{R,j}=3F_{j}l/2bh_{sp}^{2}$ where $F_{j}$ is the load coincident to a crack mouth opening displacement ($CMOD_{j})$, l is the length of the span, b is the cross-section width and $h_{sp}$ is the distance between the notch tip and the top of the cross-section.

(8) $$f_{avg}=\frac{f_{saw}\sum_{i=1}^{nsr}A_{s,i}\eta_{j,i}+\sum_{k=1}^{m}A_{k}\eta_{j,k}}{\sum_{i=1}^{nsr}A_{s,i}+\sum_{k=1}^{m}A_{k}}$$

(9) $$f_{edge}=\frac{f_{avg}}{\eta_{3D}}$$

(10) $$\tau =\frac{\tau_{raw}}{f_{edge}}$$

### 2.5. Computed Tomography Scanning and Fiber Counting

The analysis of computer tomography (CT) images has served various purposes in the concrete research field, such as the study of microstructural damage, fiber distinction, aggregate and cement paste interaction and particle distribution [54,55,56,57]. In this study, CT images were obtained from a V-TOMEX-M Micro-Computed Tomography system (GE Company) under the following configurations: 150 kV voltage, 300 μA current, 250 ms exposure time per projection, 5 frames, 0.3 mm Cu filter, 1.34 magnification, 148 μm pixel size and 1000 images. Slices alignment, beam hardening and ring artifact reduction for 3D reconstruction were carried out using Phoenix Datos-x v.2.5.0 software. Then, 3D volume was built by VGStudio max 3.0 software. CTanalyzer v.1.17.7.2 software was used for morphological quantitative analysis. Avizo Fire 8.1 software allowed for steel fiber binarization. The resulting images allowed for the evaluation of the number of fibers and their disposition in the cross section.

**Figure 8:**
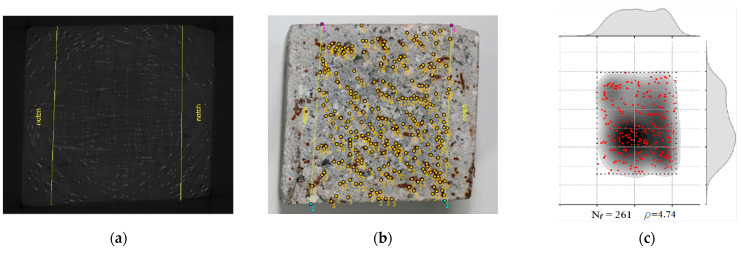
Identification of fibers of a shear cross-section: (**a**) CT image; (**b**) fiber position and counting: labels 1 and 2 are the top and bottom points, respectively, defining the limits of the shear section, and label 3 corresponds to every fiber position (**c**) distribution and density of fibers.

CT scan images such as the one presented in Figure 8a were obtained for the prisms: 6W-6, 6W-7, 6W-9, 6W-11, 8W-5, 8W-6, 8W-11, 8W-12, 9TF-2, 9TF-3, 9TF-4 and 9TF-7. CT scans of the 12 cm top flange specimens could not be obtained due to the equipment limitations of size. The position of every fiber in the shear cross-section was determined by retrieving information of both the tomography images and photos of the cross section after the test. Prisms that were not completely split during the test were sawed to take the images. ImageJ software v.1.53c [58] was employed to mark the fiber position and, consequently, count the total number of fibers (for example, the shear cross-section shown in Figure 8b). The density of fibers, ρ, is defined by $\rho =\frac{N_{f}}{b_{r}\;h}$, where $N_{f}$ is the number of fibers crossing the plane, and $b_{r}$ and h are the corresponding width and height dimensions that were measured on every prism using a Vernier caliper. The theoretical number of fibers ($n_{f,t})$ crossing a section is determined by Equation (11) [59], where A is the cross-sectional area, $A_{f}$ is the cross-sectional area of a fiber, $V_{f}$ is the volume fraction of fibers and *η* is the fiber orientation factor. To consider isotropic conditions for the fiber orientation factor, i.e., a 3D random orientation, a value of 0.405 is proposed by Soroushian and Lee [59] and a value of 0.5 is proposed by Dupont and Vandewalle [60]. Hence, the theoretical fiber density calculated is 3.16 fibers/cm^2^, considering a 3D fiber orientation (0.5) and $V_{f}=1.5\%$. Moreover, the distribution of fibers was plotted following the procedure defined in [44] only for the prism cross-sections from the prototype given the interest in knowing the distribution in the structural element. The results of an exemplary cross-section are shown in Figure 8c. In addition, the number and density of fibers of the reference prisms were determined as well.

(11) $$n_{f,t}=\eta \frac{V_{f}}{A_{f}}A$$

## 3. Results and Discussion

### 3.1. Mechanical Behavior and Effect of the Distribution and Density of Fibers

Forty-seven tests were carried out under the setup detailed in Figure 5. Seven prisms had either a relative rotation of their opposite crack surfaces or a rupture out of the notch plane and thus do not form part of the results discussed. The rest of specimens were registered in Table 3, gathered in groups that are numbered following the order in which they will be discussed in this section. The results of every prism in Table 3 are: the fiber density ρ, the maximum shear stress $\tau_{max}$, the crack width at the peak load $w_{peak}$ and the mode II fracture energy absorbed up to the failure of the specimen, considered the area under the average shear stress vs. sliding, $G_{fII}$ [61,62]. Because not all specimens reached null stress at the end of the test, the shear stress vs. sliding was extrapolated until a 4 mm slip to calculate the $G_{fII}$. Moreover, the analysis of the shear behavior followed three stages in this study: linear-elastic behavior that ended at first cracking; the response between the first crack and the achievement of the shear strength capacity; and a final softening behavior. The correlation between the fiber density and the mechanical response was treated subsequently.

#### 3.1.1. 6W Specimens

**Figure 9:**
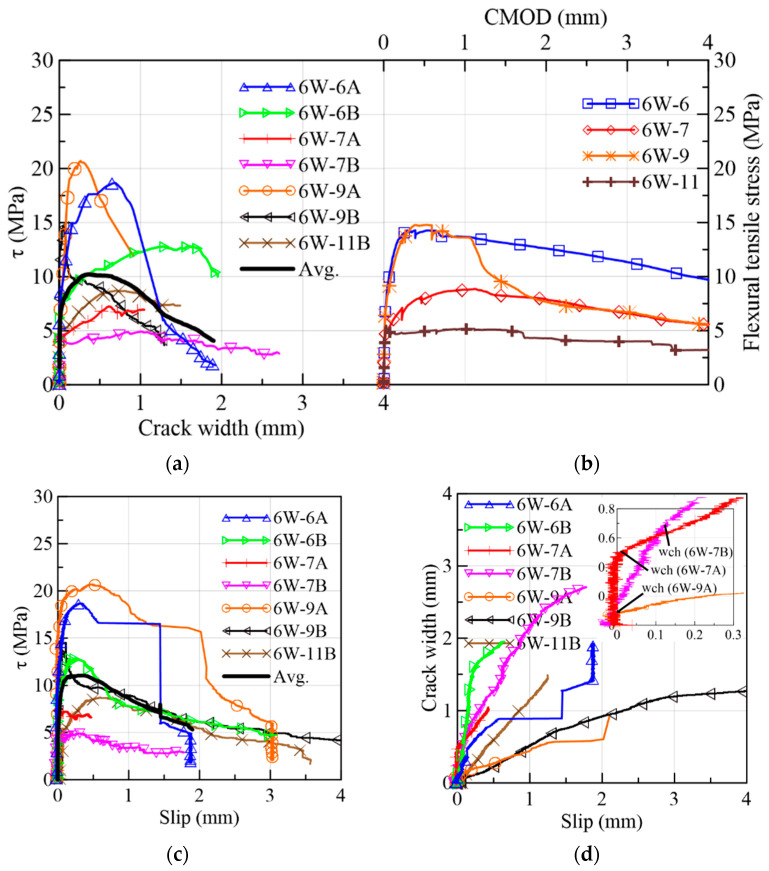
Mechanical results of 6W specimens: (**a**) shear stress vs. crack width; (**b**) flexural tensile stress vs. CMOD; (**c**) shear stress vs. slip; (**d**) crack width vs. slip.

The shear stress (τ) vs. crack width (w) of 6W prisms and the flexural tensile stress vs. crack mouth opening displacement (CMOD) from the EN14651-based tests are plotted in Figure 9a,b, respectively. On the other hand, the τ vs. slip progress is shown in Figure 9c, while Figure 9d presents the simultaneous evolution of both displacements. The first crack in the 6W prisms occurred at an average of 43% of the maximum stress. The shear stress at the cracking initiation corresponds to the point in the curves of Figure 9a when the first change in the τ/w gradient was registered. From this point up to the peak load, the gradient of shear stress transference with the increase in the crack opening decreases, resulting in a nonlinear response. The peak values for shear and flexural tensile stresses occurred for the crack width in the 0–1 mm range, with exception of 6W-6B (see Table 3 and Figure 9a,b). During the shear softening behavior, prisms presented a higher decrease in shear stress than flexural stress (see Figure 9b). This behavior could be simplified by straight lines, as will be explained in Section 3.2.

**Figure 10:**
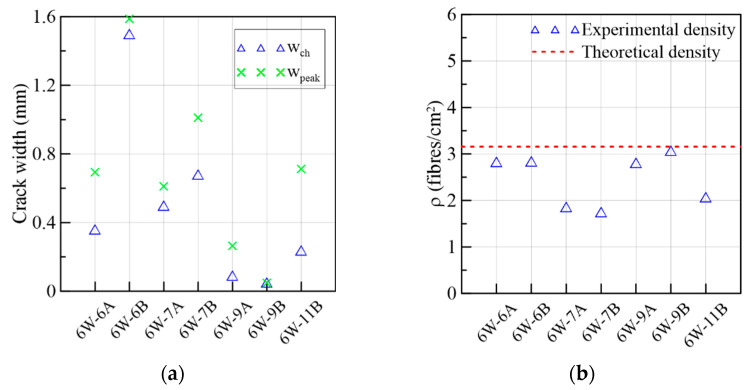
Mechanical results of 6W specimens: (**a**) crack widths at the change in behavior and at the peak load and (**b**) fiber densities.

By observing Figure 9c, the 6W specimens described a similar behavior in the ascending and descending trajectories despite the heterogeneity of the material; therefore, it was not necessary to normalize them based on the corresponding peak stresses to compare them. Comparing the average curves of the figures in crack width and slip terms, the slip was smaller than the crack width magnitude when the maximum stress was attained. In addition, Figure 9d strengthens the idea that the slip advancement was initially contained by the aggregate interlock and the fibers bridging the cracks, and, simultaneously, the crack width increased (Stage 1). However, by approaching the shear softening stage, the increase in the slip became higher than the crack width (Stage 2). The turning point where this change in behavior occurs was measured in terms of crack width ($w_{ch}$) and compared to the crack width at the peak in Figure 10a, in which $w_{ch}\;\leq w_{peak}$ for all the specimens. This two-stage behavior did not occur in the research campaigns that used specimens with a low fiber volume, where a simultaneous crack opening and slipping of a similar magnitude occurred from the start [49]. Moreover, it is important to point out that all the specimens had fiber densities lower than the theoretical density, 3.16 fibers/cm^2^, which was calculated considering a 3D random fiber orientation of 0.5 (Figure 10b).

Furthermore, no difference was found between the mechanical strength of prisms of Sides 1 (6A, 6B, 7A, 7B) and 2 (9A, 9B and 11B) of the mock-up (Figure 9a,c). Both groups gathered specimens with parameters of high and low stress values. Therefore, the stirrups of Side 2 did not interfere in the fiber and aggregate distribution. By comparing the results of Sides A and B coming from previous bending-tested prisms (6A-6B, 7A-7B and 9A-9B cases), a scatter in the curves is visible, pointing out different distributions and orientations of fibers. Although this dispersion is well noticed, there was a correspondence between the highest shear strengths (6W-6A and 6W-9A) and the highest flexural tensile strength values (6W-6 and 6W-9) in Figure 9a,b. The corresponding shear strengths of the prisms of side B, 6W-6B and 6W-9B, were lower than the values of side A while remaining higher than the shear strengths of the rest of the 6W specimens.

**Figure 11:**
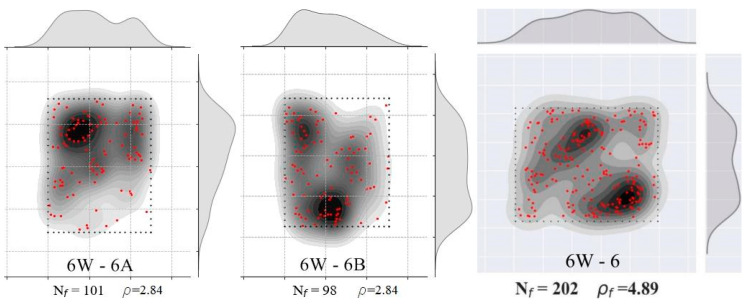

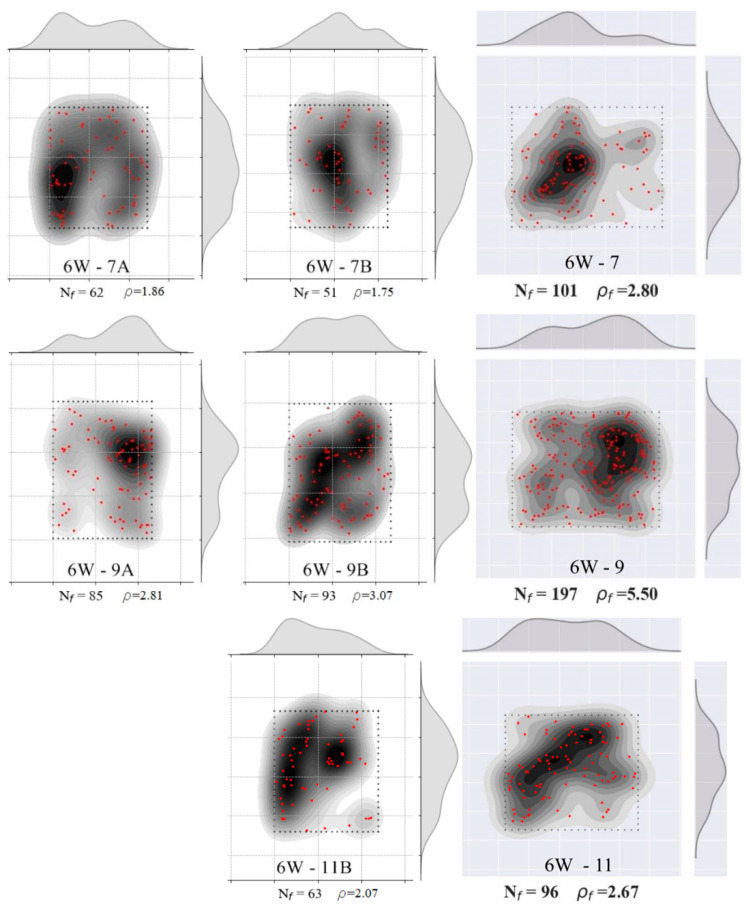
Fiber density and distribution of 6W specimens. **Left column** corresponds to prism A (shear), **middle column** corresponds to prism B (shear) and **right column** corresponds to the flexural case.

The distribution of fibers in the cross-sections was plotted in Figure 11. Counting from the left side, the first column belongs to the shear sections of prism A, the second column belongs to sections of prism B and the third column belongs to the flexural cross-sections. All of them had a random distribution of fibers, with segregation in some regions. From Table 3, it was noticed that the maximum fiber densities of the group corresponded to the specimens that also had the maximum strengths. On the Mode II fracture energy matter, a correlation between the fiber density and the energy was also found (Table 3).

#### 3.1.2. 8W Specimens

**Figure 12:**
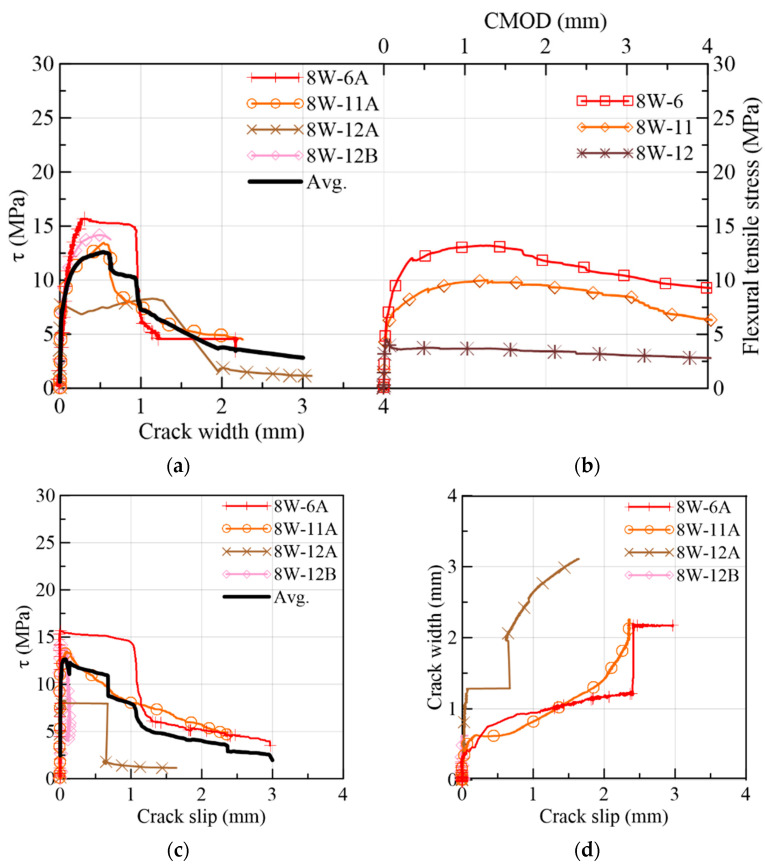
Mechanical results of 8W specimens: (**a**) shear stress vs. crack width; (**b**) flexural tensile stress vs. CMOD; (**c**) shear stress vs. slip; (**d**) crack width vs. slip.

**Figure 13:**
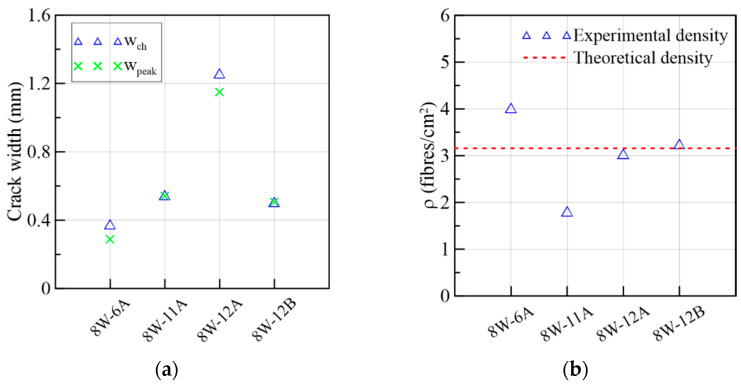
Mechanical results of 8W specimens: (**a**) crack widths at change in behavior and at peak load and (**b**) fiber densities.

The results from the direct shear and flexural tests of the 8W specimens are presented in Figure 12, following the same organization already adopted for the 6W specimens. As Figure 2a,b show, 8W-6 belonged to Side 1 of the web’s mock-up, while 8W-11 and 8W-12 were extracted from Side 2 of the web. By comparing the responses in crack width and slip terms (Figure 12a,c), there was no remarkable difference between Sides 1 and 2. The shear stress at crack initiation was, on average, 58% of the shear strength (Figure 12a). The 8W curves strengthen the results’ trend reported for the 6Ws by indicating that the fiber volume (1.5%) was able to produce a gain of strength after the first crack stress, describing a nonlinear behavior between the first crack and the peak load. Test results found elsewhere [26] proved that the low volume of fibers reinforcing a conventional concrete matrix was not enough for ensuring the aforementioned shear hardening phase. By comparing the peak stresses of the shear and flexural results in Figure 12a,b, a correspondence was found between them: 8W-6A and 8W-6 had the maximum stresses at peak loads, and, in the opposite case, 8W-12A and 8W-12 had the minimum. Furthermore, the corresponding average crack width at a peak load of 8Ws was approximately 0.5 mm for the shear test and about 1 mm for the flexural test. Moreover, some prisms presented a plateau in the τ vs. slip response after the peak load (8W-6A and 8W-12A in Figure 12c). This plateau is a representation of the load maintenance just before the rupture of the shear cross-section. Slip displacements until the peak load were lower than the crack widths (Figure 12c,d), but this tendency was gradually inverted during the cracking process, as already observed in the 6W specimens. The crack width ($w_{ch}$) when this change in behavior occurred was compared to the crack width at the shear strength $\left(w_{peak}\right)$ in Figure 13a, from which it can be inferred that $w_{ch}\;\sim w_{peak}$. It is also important to identify that the experimental fiber densities of these specimens were around or higher than the theoretical fiber density (3.16 fibers/cm^2^), with the exception of 8W-11A (Figure 13b).

**Figure 14:**
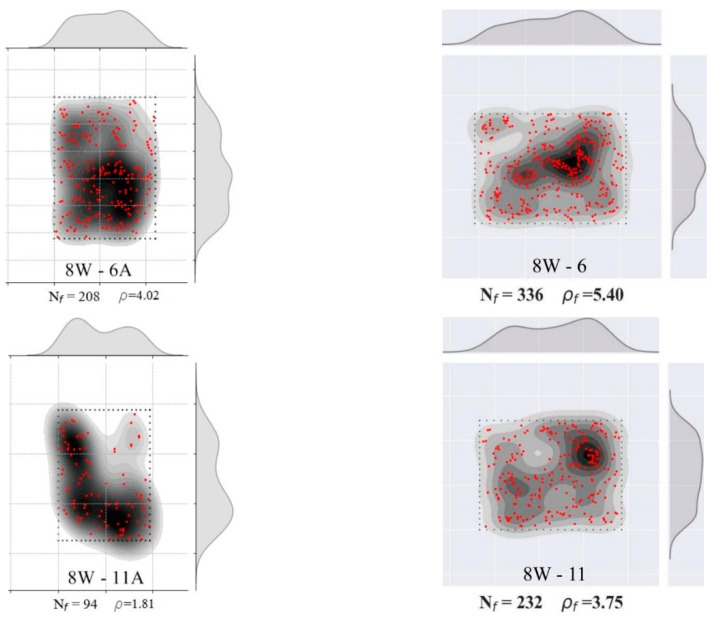

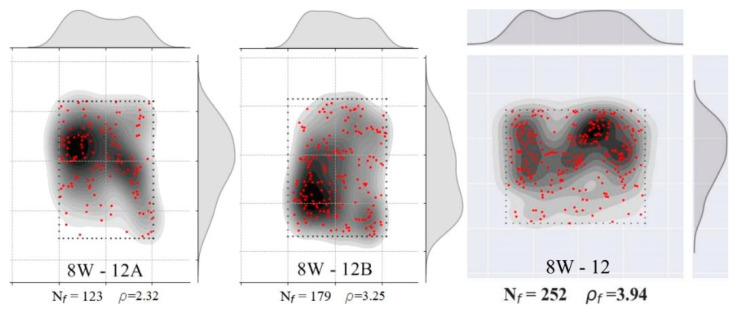
Fiber density and distribution of 8W specimens. **Left column** corresponds to prism A (shear), **middle column** corresponds to prism B (shear) and **right column** corresponds to the flexural case.

The fiber distribution in the 8W cross-sections was grouped in Figure 14, where a non-uniform fiber distribution is visible. As in the 6W case, the prisms A and B were placed in the first two columns, and the flexural sections were placed in the third one. The maximum fiber density of 8W-6A (see Group 2 in Table 3) has a correspondence with the maximum values of shear strength and fracture energy. On the other hand, the minimum fiber density (8W-11A) produced the second-lowest shear strength.

The fracture energy of 8W-12B (1.99 N/mm) was the lowest in the group. This particular result indicates that the orientation of fibers and the arrangement of aggregates at the fracture plane might have been not so favorable for mobilizing the potential resisting mechanisms of the relatively high fiber density (3.25 fibers/cm^2^) registered in this specimen.

#### 3.1.3. 6R-8R Reference Specimens

**Figure 15:**
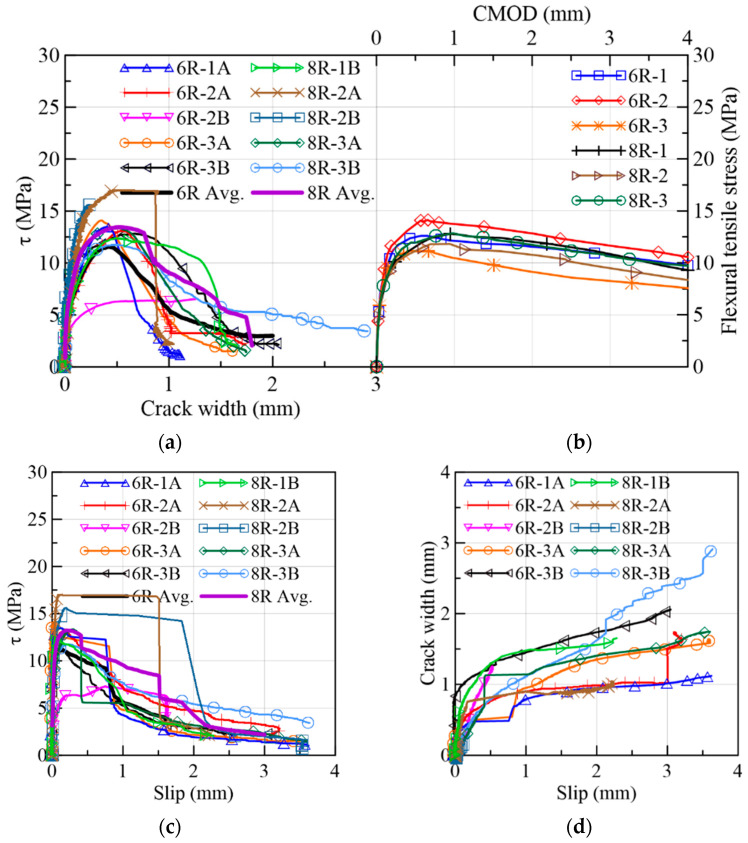
Mechanical results of 6R and 8R specimens: (**a**) shear stress vs. crack width; (**b**) flexural tensile stress vs. CMOD; (**c**) shear stress vs. slip; (**d**) crack width vs. slip.

**Figure 16:**
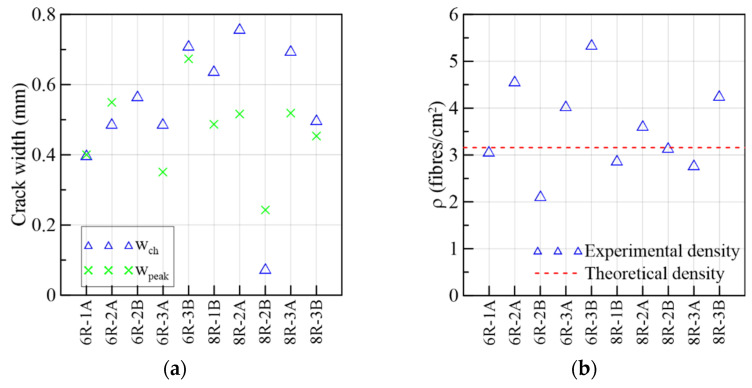
Mechanical results of 6R and 8R specimens: (**a**) crack widths at change in behavior and at peak load and (**b**) fiber densities.

The results of the reference prisms 6R and 8R were gathered in Figure 15 and in the Group 3 of Table 3 not only to be compared to the specimens from the webs of the prototype but also because they share similarities in stress range, crack widths and slips. The mechanical behavior is described in terms of τ vs. crack width in Figure 15a, the flexural results in Figure 15b, the sliding progress (τ vs. slip) in Figure 15c, crack width vs. slip in Figure 15d, crack widths in Figure 16a and fiber densities in Figure 16b. Overall, a smaller dispersion was found in these curves compared to their counterparts coming from the webs. Certainly, the method of pouring fresh concrete is better controlled in a small prism than in real structures. This strengthens the importance of building a mock-up to determine the actual mechanical response of the structural element. In Figure 15a, it is observed that the first crack occurred at 37%, on average, of the maximum stress. As the load increased, the curves described a nonlinear behavior between the first crack and the maximum load. Due to a problem with the clip gauge in the 6R-2B, the monitoring cracking process was not able to record completely. It can be seen in Figure 15c that a plateau is formed after the peak load preceding the failure of the 6R-1A, 6R-3A, 8R-2A and 8R-2B specimens.

In the 6R group, the maximum fiber densities belonged to 6R-3B and 6R-2A. From Table 3, it can be seen that the maximum stresses corresponded to 6R-3A and 6R-1A. This means that the shear stress capacity is not only controlled by the number of fibers crossing the shear plane. The orientation of fibers defines the efficiency of their contribution to the maximum shear stress [25]. The fiber orientation will be obtained in the continuation of this research. According to the fiber densities and shear strength parameters of the 8R specimens in Table 3, it is concluded that high fiber densities (8R-2A, 8R-2B) correlated well with the shear stress capacity, although the highest fiber density (8R-3B) did not cause the highest strength nor fracture energy among the 8R prisms. A poor arrangement of aggregates and orientation of fibers might be the reason for this performance of 8R-3B.

#### 3.1.4. Top-Flange Specimens of the Transverse or X-Axis Direction

**Figure 17:**
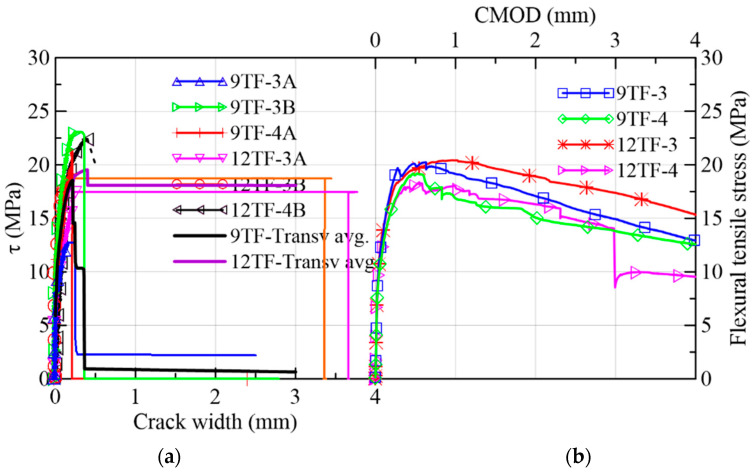

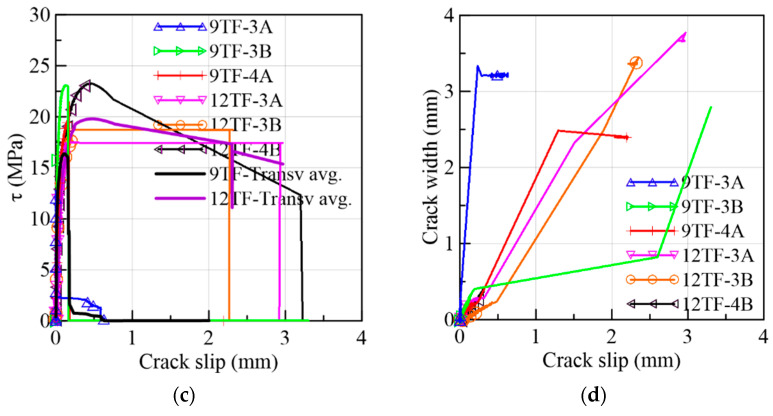
Mechanical results of 9TF-12TF specimens of the transverse direction: (**a**) shear stress vs. crack width; (**b**) flexural tensile stress vs. CMOD; (**c**) shear stress vs. slip; (**d**) crack width vs. slip.

**Figure 18:**
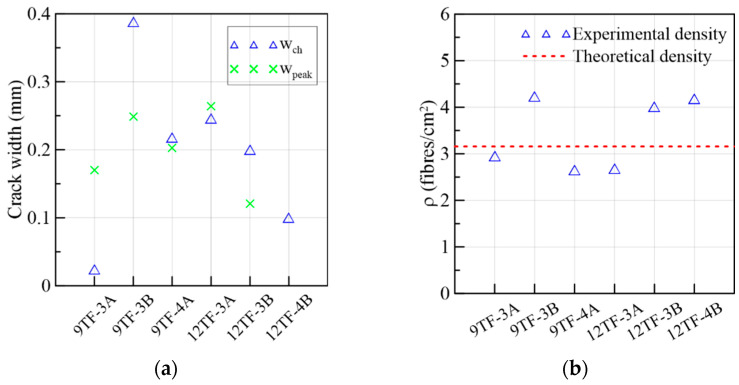
Mechanical results of 9TF-12TF specimens of the transverse direction: (**a**) crack widths at the change in behavior and at the peak load and (**b**) fiber densities.

**Figure 19:**
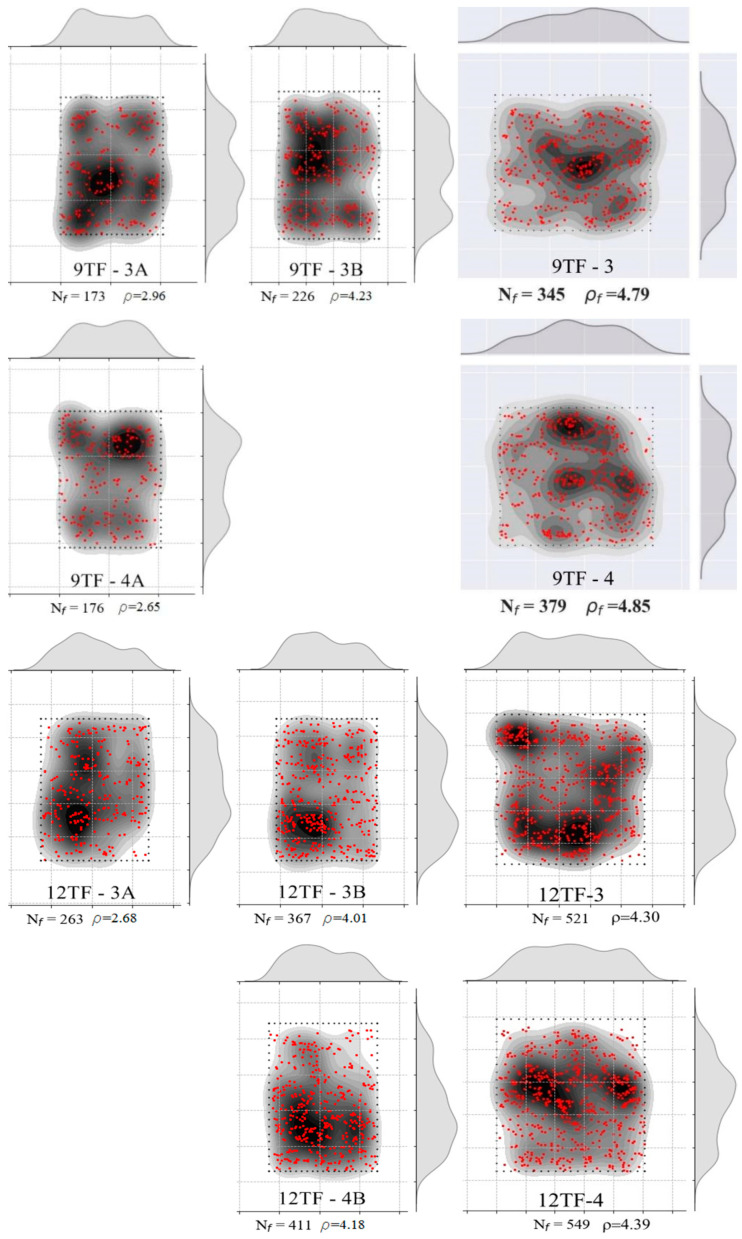
Fiber density and distribution of top-flange specimens of the transverse direction. **Left column** corresponds to prism A (shear), **middle column** corresponds to prism B (shear) and **right column** corresponds to the flexural case.

**Figure 20:**
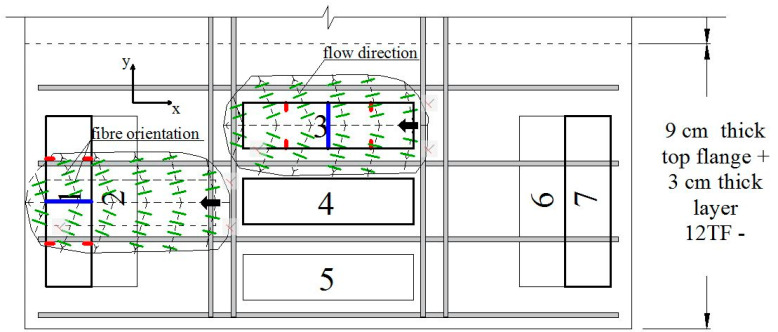
Fiber orientation in the top flanges of the R/SFRC box girder prototype.

**Figure 21:**
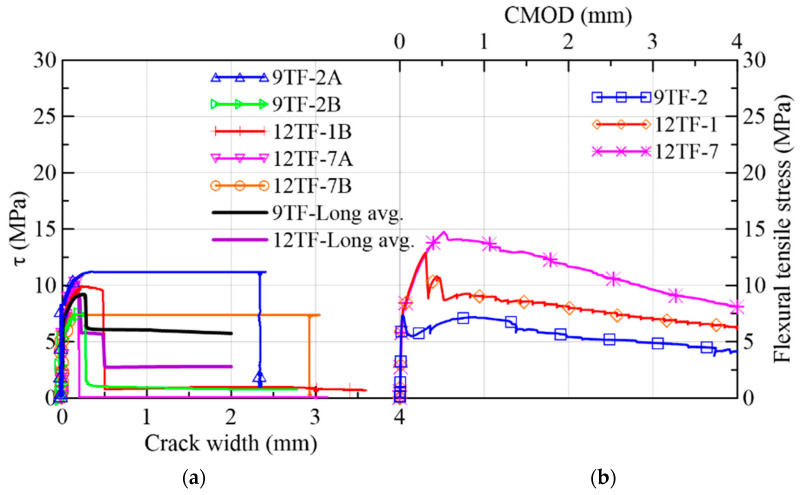

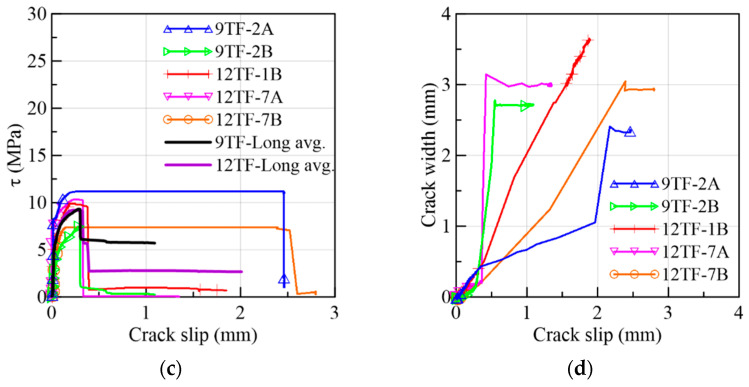
Mechanical results of 9TF-12TF specimens of the longitudinal direction: (**a**) shear stress vs. crack width; (**b**) flexural tensile stress vs. CMOD; (**c**) shear stress vs. slip; (**d**) crack width vs. slip.

**Figure 22:**
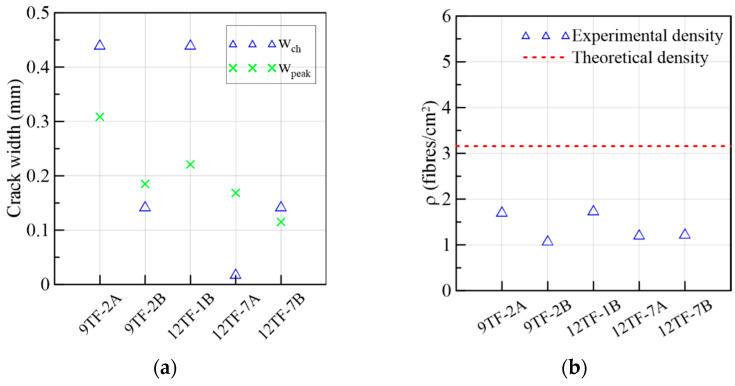
Mechanical results of 9TF-12TF specimens of the longitudinal direction: (**a**) crack widths at the change in behavior and at the peak load and (**b**) fiber densities.

**Figure 23:**
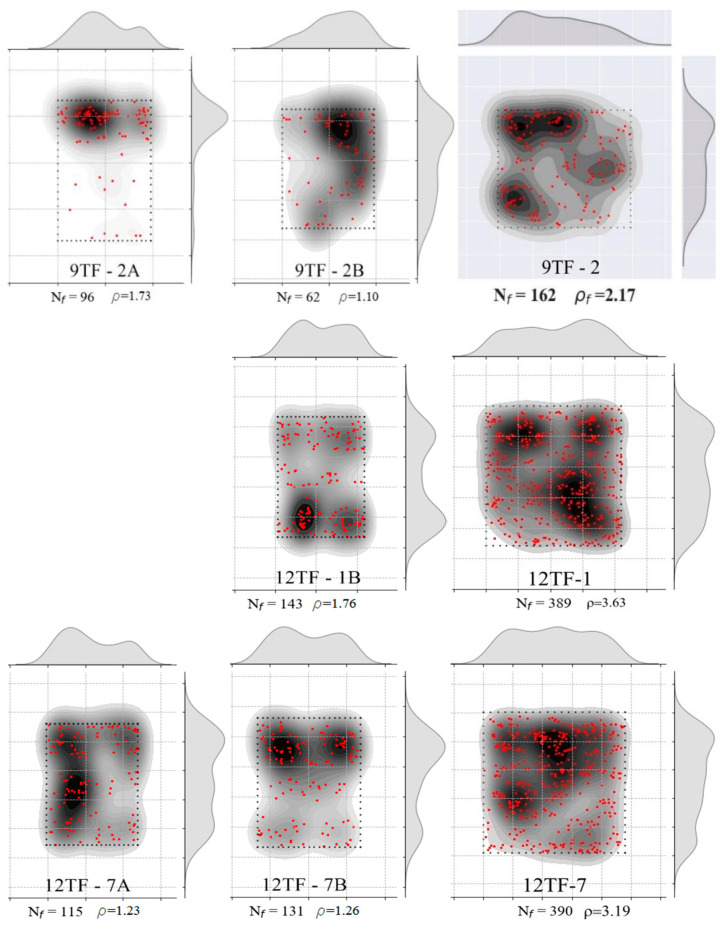
Fiber density and distribution of top-flange specimens of the longitudinal direction. **Left column** corresponds to prism A (shear), the **middle column** corresponds to prism B (shear) and the **right column** corresponds to the flexural case.

For the discussion of the results, the top flange prisms were separated according to the direction of their longitudinal axis with respect to the longitudinal axis of the box girder prototype which corresponds to the *y*-axis in Figure 2c. The prisms whose longitudinal axis is orthogonal to the *y*-axis (9TF-3A, 9TF-3B, 9TF-4A, 12TF-3A, 12TF-3B and 12TF-4B) are grouped as prisms of transverse direction (*x*-axis). Their test results are discussed first and are presented in Figure 17, Figure 18, Figure 19 and Figure 20 and in Group 4 of the results in Table 3. On the other hand, the prisms whose longitudinal axis is parallel to the *y*-axis (9TF-2A, 9TF-2B, 12TF-1B, 12TF-7A and 12TF-7B) are grouped as prisms of longitudinal direction (*y*-axis) and are discussed subsequently (Figure 20, Figure 21, Figure 22 and Figure 23 and Group 5 of Table 3). From Table 3, it is evidenced that the ranges of fiber density and the maximum stress of the prisms of transverse direction were 2.65–4.23 fibers/cm^2^ and 12.74–23.27 MPa, respectively, whereas the same ranges for the longitudinal-direction prisms were 1.1–1.76 fibers/cm^2^ and 7.40–11.20 MPa, respectively. After analyzing the position of the shear planes of both groups in Figure 2c, the ranges of fiber density and the shear stress capacity already mentioned, it is concluded that there was an alignment of the steel fibers in the *x*-axis direction (i.e., transverse direction to the longitudinal axis of the box girder prototype). It can be inferred that the fibers aligned orthogonally to the flow direction of fresh concrete, as was found elsewhere [24] and is exemplified for the 12 TF prisms in Figure 20, where the green lines represent the fibers and the black dashed lines represent the flow direction. In Figure 20, 12TF-3 is chosen as an exemplary prism of the group of the transverse direction, and 12TF-1 is chosen as an example of the longitudinal direction group. For the former, fibers tend to have more efficient fiber orientation factors crossing the shear and flexural sections compared to the latter.

Having established the influence of the SFRC flow direction on the mechanical response of both top-flange groups of specimens, the results of the prisms of the transverse direction are discussed first. The first crack of these prisms opened, on average, at 40% of the maximum stress (Figure 17a). The average crack width at the peak load for shear and flexure (Figure 17b) was 0.20 and 0.67 mm, correspondingly. A sudden drop of strength was experienced, which was correlated with an abrupt split of the specimens. A plateau was formed immediately after the peak in 12TF-3A and 12TF-3B. The failure was characterized by being sudden. Figure 19 presents the distribution of fibers across the shear planes, which presented less segregation of the fibers compared to the web cases (Figure 11 and Figure 14). The performance in τ vs. slip terms (Figure 17c) and crack width vs. slip terms (Figure 17d) indicates that the sliding is controlled until values belonging to the maximum stress. Afterwards, the failure and complete split of the prisms occurred. In Figure 18a, mixed responses of $w_{ch}$ with respect to $w_{peak}$ were found. The 12TF-4B had a drop in the clip-gauge; thus, its $w_{peak}$ could not be registered. In addition, these prisms presented experimental fiber densities lower and higher than the theoretical density (Figure 18b).

#### 3.1.5. Top Flange Specimens of the Longitudinal or *Y*-Axis Direction

The results of the top flange specimens whose longitudinal axis is parallel to the longitudinal axis of the prototype (*y*-axis in Figure 2c) are gathered in Figure 21 and in Group 5 of Table 3. The range of peak stresses and fiber densities is lower than the range of the group of the transverse direction, as can be verified in Table 3 and was detailed before. By comparing the fiber distribution of the top-flange prism sections of the transverse and longitudinal directions (Figure 19 and Figure 23, respectively), it was observed that there was not only segregation in the latter group, but the reinforcement bar crossing the prisms near the shear cross-sections (Figure 2c) might have also complicated a more uniform distribution. The average first crack stress represented 64% of the shear strength (Figure 21a). The slip at the maximum stress had a similar magnitude as the corresponding crack width (Figure 21a,c), which means that the low number of fibers crossing the shear cross-section are not enough to control the slip seen in the other prism sets. A plateau in 9TF-2A and 12TF-7B occurred before the specimens experienced a complete split.

From Figure 21d and Figure 22a, it can be seen that the crack width at the turning point (*w_ch_*) occurs before 0.5 mm, and, subsequently, a rapid increase in either the crack width (12TF-7A and 9TF-2B) or a combination with the increase in the slip (9TF-2A, 12TF-1B and 12TF-7B) were found. All cases experienced a complete separation of the specimen. By comparing *w_ch_* and *w_peak_*, mixed relations were found, and considering that the experimental fiber densities were lower than both the theoretical density (Figure 22b) and the fiber densities of the other prism sets, the contribution to the shear transfer mechanism is reduced compared to the rest of the prims. These low fiber density values occurred given that the fibers aligned in the *x*-axis direction of Figure 2c, as detailed in Section 3.1.4. The 12TF-1 example in Figure 20 presents a less efficient fiber orientation and, consequently, fewer fibers crossing their cross-sections compared to the opposite case of top-flange specimens. It is noticed that when the prisms had a more efficient fiber orientation, their fiber densities were around or higher than the theoretical density (Figure 18b). The opposite occurs with prisms of a poor fiber orientation.

#### 3.1.6. 9R-12R Reference Specimens

**Figure 24:**
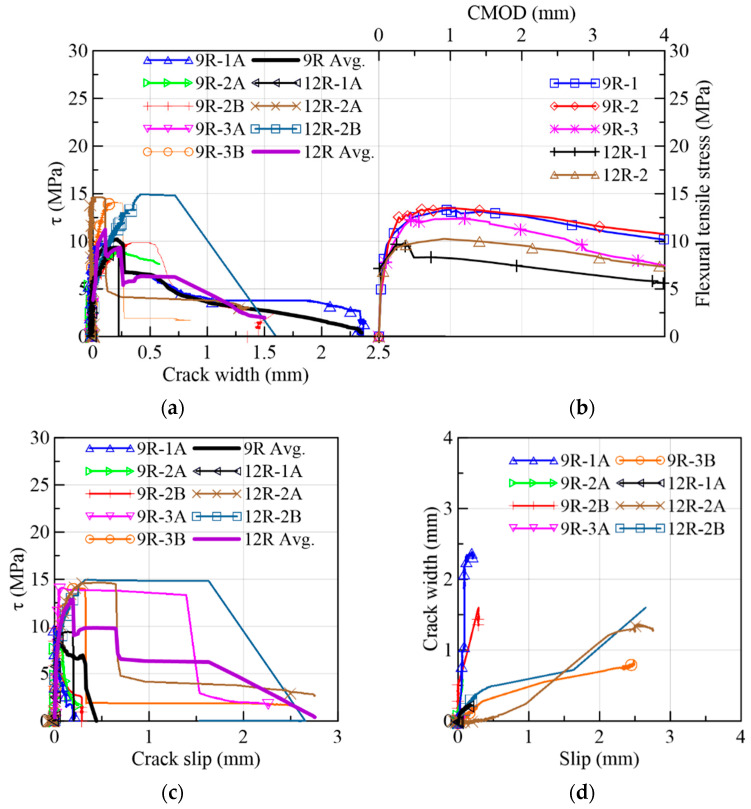
Mechanical results of 9R and 12R specimens: (**a**) shear stress vs. crack width; (**b**) flexural tensile stress vs. CMOD; (**c**) shear stress vs. slip; (**d**) crack width vs. slip.

The results of the 9R-12R specimens, the counterpart of the top flange specimens, were gathered in Figure 24 and in Group 6 of Table 3. The τ vs. crack-width responses are represented in Figure 24a, from which it was found that the average first-crack stress/shear strength ratio was 34%, which, in turn, is comparable to the result of the top-flange transverse-direction prisms (40%) but distant from the top-flange longitudinal-direction case (64%). This demonstrates how much the alignment of fibers can substantially influence their mechanical contribution to the shear transfer mechanism. Two clip-gauges dropped (9R-2A and 9R-3A) during the cracking process. The average crack widths at the peak load for the shear and flexure were 0.25 mm and 0.87 mm, correspondingly (Figure 24a,b). The flexural cases presented less dispersion than the shear results. By analyzing the results in τ vs. slip terms (Figure 24c), it was determined that the 9R-12R set had more dispersed curves than the 6R-8R case (Figure 15c) but similar curves to the top-flange cases (Figure 17c and Figure 21c). In addition, 9R-3A, 12R-2A and 12R-2B described a plateau before the strength drop (Figure 24c). Despite being specimens that were cast in molds, the dispersion between prisms A and B remained.

**Figure 25:**
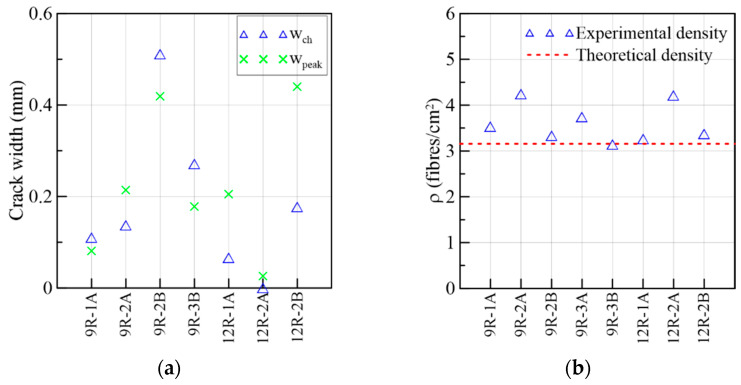
Mechanical results of 9R and 12R specimens: (**a**) crack widths at the change in behavior and at the peak load and (**b**) fiber densities.

On the crack-width vs. slip relation (Figure 24d), there is a Stage 1 where the slip is controlled by the shear mechanism, and at a Stage 2, two different results were found: the first one consists of a complete crack opening (9R-1A and 9R-2B), and the second one consists of a major increase in the slip (9R-3B, 12R-2A and 12R-2B). In Figure 25a, the comparison between the crack width at which the crack width–slip relation changes from Stage 1 to Stage 2 ($w_{ch}$) and the crack width at the peak load $\left(w_{peak}\right)$ presented mixed results despite the fact that most of the fiber densities are above the theoretical density in Figure 25b (3.14–4.25 fibers/cm^2^). The drop in the clip gauge of 9R-3A occurred at an early loading stage; thus, it is not included in Figure 25a.

#### 3.1.7. Summary of the Analysis of the Shear Behavior

The different sets were gathered in four groups: 6W-8Ws (W), 6R-12Rs (R), top flanges of the longitudinal direction (TF-L) and top flanges of the transverse direction (TF-T); then, average values and variation coefficients were obtained to summarize the mechanical parameters discussed previously. They are presented in Table 4. According to the first crack stress/strength ratios, it can be emphasized that the contribution of fibers to the increase in strength after the first crack stress varied mainly in terms of the pouring methods of every group. This gain of strength was higher in the reference specimens than in the mock-up prisms due to the pouring of concrete being better controlled in small molds; thus, this reinforces the importance of testing extracted specimens from a full-scale prototype. On the maximum stress parameter, it is remarkable that an alignment of fibers in the TF-T group produced not only high fiber density values but also a more efficient fiber orientation than the rest of the groups and, consequently, the highest average shear strength. The reference specimens also had high fiber densities; however, it can be assumed that they had less efficient fiber orientations. The opposite case to TF-T in terms of shear strength was the TF-L set. The influence of these differences among the prism groups can be extended to the average fracture energies registered in Table 4. The values of the crack width at the maximum stress in Table 4 allow for the inference that the peak load occurs at a lower crack width in the 9 cm and 12 cm prisms of the prototype compared to the prisms of 6 cm and 8 cm in size. This behavior also occurs in the reference specimens (see the average crack widths in Figure 15a and Figure 24a) About the $w_{ch}/w_{peak}$ ratios, when the fiber density augments, the point of the change in the behavior ($w_{ch}$) is approximate to $w_{peak}$ or can even be higher. This is confirmed by comparing the fiber densities in the W, R and TF-T prisms (Figure 9, Figure 12, Figure 15 and Figure 17) and their corresponding average $w_{ch}/w_{peak}$ ratios in Table 4. Since the TF-L set presents mixed $w_{ch}/w_{peak}$ ratios, no further conclusions can be drawn. In the end, these results strengthen the importance of building a mock-up to know the mechanical response of the structural element and provide a proper design.

#### 3.1.8. Correlation between Fiber Density and Mechanical Parameters

**Figure 26:**
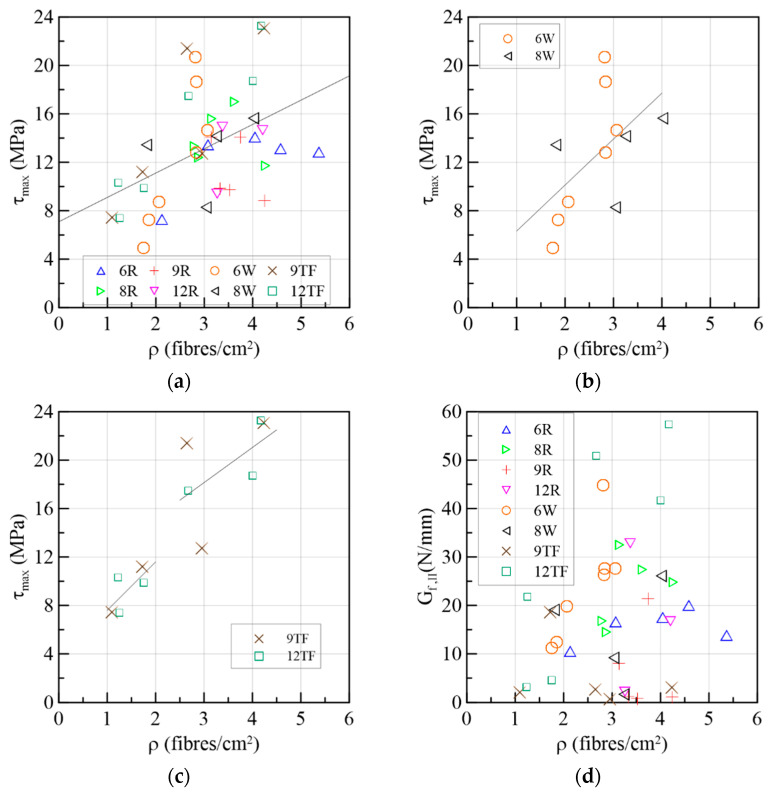
Correlation between fiber density and mechanical parameters: (**a**) shear stress capacity of all prisms; (**b**) shear strength of web specimens; (**c**) shear strength of top flange specimens; and (**d**) Mode-II fracture energy absorbed up to the failure of the specimen.

In Figure 26a, the relation between the fiber density and shear strength of all the specimens was carried out by establishing a linear regression. Overall, there was a proportional relationship between the variables, although scattering is present. Considering that the batching method influences the mechanical performance, the results of the extracted specimens were separated from the entire group, 6W-8Ws in Figure 26b and 9TF-12TFs in Figure 26c. On the top flange cases (Figure 26c), there were two groups with a good and poor alignment of fibers, as explained previously. The gradients of each linear regression in Figure 26b,c are similar. On the other hand, the 6R-8R-9R-12R results do not present this trend (see Figure 26a) because of the difference in the batching conditions and, consequently, the fiber orientation between the extracted and reference prisms. In the extracted specimens, the SFRC was poured in molds of the full-scale prototype model, and the flow was either oriented due to gravity (webs) or man-made oriented (top flanges). In the reference cases, the SFRC was poured from an extremity of each mold, and the flow was limited by the small dimensions of the molds. In addition, a relation between the Mode II fracture energy and fiber density was sought, but, as shown in Figure 26d, it was not observed. In a future work, the prisms will be classified in clusters according to their fiber orientation, considering that the efficiency of the mechanical response of the fiber also depends on this factor.

### 3.2. Shear/Flexural Tensile Stress Ratio versus Crack Opening and Normalized Crack Dilatancy Law

The search of a relationship between shear and flexural tensile stresses was based on three motives. First, the scarcity of studies relating shear results with other mechanical characterization tests is a current research gap. In the case of steel fiber-reinforced concrete, it is important to establish the relation with the tensile properties considering the major contribution of fibers in this field. The EN14651 flexural test is one of the most widely accepted standards in the research community for indirectly determining the tensile properties of the material. Thus, if a bridge is created between both results, the shear behavior could be estimated from the parameters originated in flexural tests. A second motive relies on the fact that shear tests are more difficult to carry out than flexural tests. For the former, instrumentation is needed to control two simultaneous displacements, and, at the same time, other effects could influence the results, such as the relative rotation of crack surfaces in the FIP case, the flexural and arch action that can occur in the JSCE case and so on. Again, in the FIP case, the positioning of non-symmetric loads requires additional attention, and the available test setup cannot meet the required load spacing. Conversely, the EN14651 test requires less instrumentation to measure the crack opening at a half span, and loads are placed symmetrically (one acting load at a half span and two support loads). The third motive is the relevancy of establishing this relation from specimens extracted from real structural elements, avoiding the prisms molded with specific dimensions. The previous section proved the differences in mechanical performances despite the corrections of sawing and the wall effect. The flow of fresh concrete in real structures cannot be reproduced without using same-scale prototypes. Therefore, the possibility of obtaining this relation from a mock-up of a box girder strengthened the applicability of the results.

**Figure 27:**
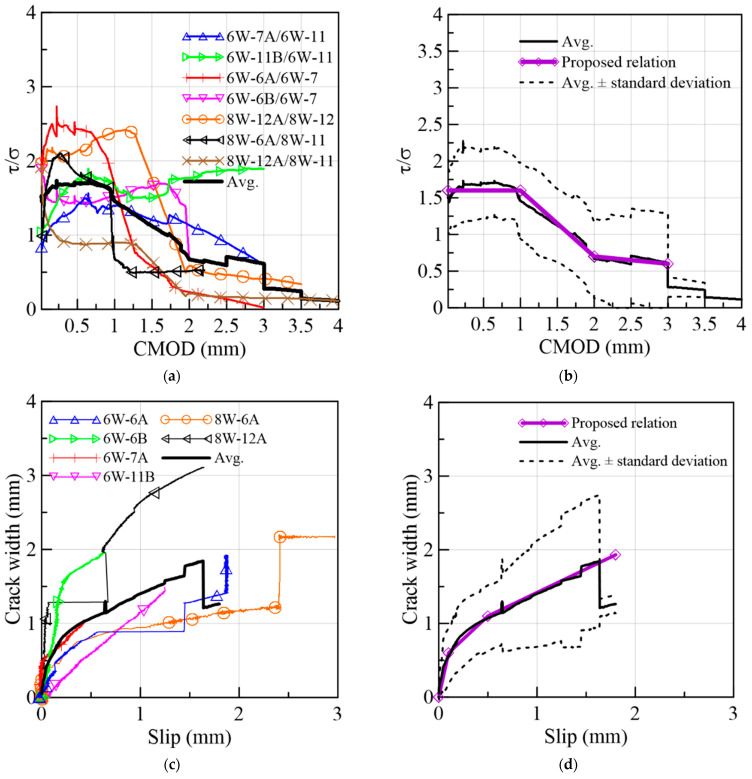
Shear/flexural tensile stress ratios vs. CMOD from (**a**) selected specimens, (**b**) proposed crack dilatancy law, (**c**) crack width–slip relation and (**d**) proposed crack width–slip relation.

To compare the shear and flexural behavior in terms of stress and crack width, curves of shear stress/flexural tensile stress ratios vs. CMOD were selected. This normalization of the crack dilatancy response, τ vs. crack width, is relevant if the shear and flexural planes are mechanically equivalent, i.e., they come from the same concrete matrix and have similar fiber densities. In addition, it was intended that this normalization be carried out based on results that are representative of the common SFRC batching in the construction industry; therefore, the 6W and 8W groups of specimens were chosen because they have a top-to-bottom pouring procedure. So, the fracture planes under comparison came from different prisms considering the heterogeneity of the distribution of fibers, even in the same prism. Figure 27a presents the experimental pairs formed in addition to their average normalized curve. It can be seen that when 0≤CMOD≤1, the stress ratio varied between 1 and 2.5, and when 1≤CMOD≤2, the ratio fell to the range of 0.25–2, this being coincident with steep drops in strength in the shear prisms. When 2≤CMOD≤3, the ratios were in the 0–1.25 range and presented a less steep decline than the previous interval. The average relation is depicted in Figure 27b, along with two curves (top and bottom) representing the standard deviation. Subsequently, a trilinear relation describing this behavior could be proposed in Figure 27b, it being composed of three segments describing the average experimental response detailed. Equations (12)–(14) define the relation.

(12) $$\begin{array}{l} \tau /\sigma =1.6\;\;\;\;\;\;\;\;\;\;\;\;\;\;\;\;\;\;\;\;\;\;\;\;\;\;\;\;\;\;\;\;\;\;\;\;\;\mathrm{for}\;0\leq w\leq 1.0 \end{array}$$

(13) $$\begin{array}{l} \tau /\sigma =-0.9\cdot CMOD+2.5\;\;\;\;\;\;\;\;\mathrm{for}\;1.0\leq w\leq 2.0 \end{array}$$

(14) $$\begin{array}{l} \tau /\sigma =-0.1\cdot CMOD+0.9\;\;\;\;\;\;\;\;\mathrm{for}\;2.0\leq w\leq 3.0 \end{array}$$

In addition, a relation between the crack width (w) and slip (s) was established from the results of the same set of prisms, as shown in Figure 27c. The first stage (s≤0.1 mm) is characterized by a stiffer slip response than the subsequent stages, representing the influence of fibers on the cracking process. The second stage (0.10≤s≤0.50 mm) had a lower gradient than the first one, indicating the advancement of the pullout of fibers and the increase in slip despite the aggregate interlock. Finally, a third stage was defined by the advanced stage of the fiber pullout or a rupture of fibers and a decrease in the aggregate interlock resistance. After s>1.80 mm, there were not enough experimental curves to obtain the average tendency. Therefore, the proposed relation which follows the average w–s response (Figure 27d) was defined, as shown in Equations (15)–(17). As the slip continues increasing, it is expected that the w–s relation approximates a horizontal asymptote, i.e., the crack width achieves a maximum value which is a multiple of the maximum aggregate size.

Thus, the experimental shear behavior of an SFRC could be estimated from EN14651-based flexural results by following the relations proposed.

(15) $$\begin{array}{l} w=6\cdot s\;\;\;\;\;\;\;\;\;\;\;\;\;\;\;\;\;\;\;\;\;\;\;\;\;\;\;\;\;\;\;\;\;\;\;\;\;\;\;\;\;\;\;\;\;\;\;\;\;\;\;\;\;\mathrm{for}\;0\leq s\leq 0.10 \end{array}$$

(16) $$\begin{array}{l} w=1.25\cdot s+0.48\;\;\;\;\;\;\;\;\;\;\;\;\;\;\;\;\;\;\;\;\;\;\;\;\;\;\;\;\;\;\;\;\;\;\;\;\;\mathrm{for}\;0.10\leq s\leq 0.50 \end{array}$$

(17) $$\begin{array}{l} w=0.64\cdot s+0.78\;\;\;\;\;\;\;\;\;\;\;\;\;\;\;\;\;\;\;\;\;\;\;\;\;\;\;\;\;\;\;\;\;\;\;\;\;\mathrm{for}\;0.50\leq s\leq 1.80 \end{array}$$

## 4. Conclusions

The experimental study of the crack propagation when shear loads were applied to prismatic specimens extracted from an SFRC full-scale box girder prototype allowed for the establishment of the following conclusions:

1. A normalized crack dilatancy law, τ/σ vs. CMOD, was proposed, which allowed for the determination of a relation with flexural tests carried out under the EN14651 setup, while acknowledging the significant dispersion of the experimental results. In addition, a crack width–slip relation was proposed, which can complete the estimation of the shear behavior from the flexural results. This is advantageous considering that the flexure test is less instrumented and easier to execute than a shear test setup and is also available for non-wealthy laboratories. Given that the batching method influences the mechanical response of SFRC, this bridge between standardized tests is additionally relevant by being based on the experimental results of the extracted prisms of a prototype.
2. The dispersion of the shear mechanical properties in a structural element can only be known by performing tests on specimens extracted from a prototype. The scatter of results occurred not only between points of extraction but also between two halves of the same specimen extracted. This study acquires value given that it quantitatively presents the variability of mechanical performance in a structural element. It strengthens the ideas that SFRC is a heterogenous material; thus, prototype construction is highly recommended to determine the actual mechanical response.
3. The alignment of fibers perpendicular to the flow direction of fresh concrete was confirmed by the results of top-flange prisms. In addition, when fiber density increases, the point of the change in behavior from a controlled slip to a significant increase in it can be delayed.
4. The batching method highly influenced the fiber density and the fiber orientation in the cross-sections. A proportional relation between the fiber density and the shear strength was found for the specimens extracted from the prototype model, but not for the reference specimens. Mode II fracture energy absorbed up to failure vs. fiber density did not display a clear trend. In a future work, the fiber orientation will be obtained, and inverse analysis will be carried out for the tests performed. The obtention of the fiber orientation will allow for the classification of the prisms in clusters according to the efficiency of the mechanical response of the fiber; thus, it is expected that the dispersion of results can be reduced.

## Figures and Tables

**Table 1:** Material proportions [44].

Materials	Proportions (kg/m^3^)
Coarse aggregate (d < 9.5 mm)	454.0
River sand	830.6
Sieved river sand (d < 0.85 mm)	100.0
Crushed Silica (Mesh No. 325)	70.0
Cement CP III 40	360.0
Fly ash	168.0
Silica fume	45.0
Water	180.0
Dramix 65/35 BG Steel fibers	117.0
Superplasticizer (% cement weight)	8.0
Viscosity modifying agent (% cement weight)	0.1

**Table 2:** Details of prismatic specimens.

Cross-Sections (cm^2^)		Spans, *D* (cm)		Labels of Specimens	
Full (b·h)	$\mathrm{Notched}\;(b_{r}\cdot h)$	Top	Bottom	Mock-Up	Reference
6 × 6	4 × 6	6	6	6W-XY	6R-XY
8 × 8	5 × 8	6.5	6.5	8W-XY	8R-XY
9 × 9	6 × 9	7	7	9TF-XY	9R-XY
12 × 12	8 × 12	6.5	9	12TF-XY	12R-XY

**Table 3:** Results of shear tests.

				At Peak		$G_{fII}\;(N/\mathrm{mm})$
Group	Prism	Details *	ρ	$\tau_{max}\;(\mathrm{MPa})$	$w_{peak}\;(\mathrm{mm})$	
1	6W-6A	NRR	2.84	18.66	0.69	26.33
	6W-6B	NRR	2.84	12.82	1.59	27.60
	6W-7A	NRR	1.86	7.23	0.61	12.39
	6W-7B	RRE	1.75	4.91	1.01	11.18
	6W-9A	RRE	2.81	20.70	0.26	44.81
	6W-9B	NRR	3.07	14.65	0.05	27.57
	6W-11B	RRE	2.07	8.72	0.71	19.77
2	8W-6A	NRR	4.02	15.66	0.29	26.08
	8W-11A	NRR, PF	1.81	13.45	0.54	19.07
	8W-12A	NRR	3.04	8.28	1.15	9.23
	8W-12B	NRR, PF	3.25	14.16	0.51	1.72
3	6R-1A	NRR	3.08	13.46	0.40	16.63
	6R-2A	SRR, PF	4.58	13.13	0.55	20.02
	6R-2B	NRR, PF	2.13	7.30	-	10.50
	6R-3A	NRR	4.05	14.10	0.35	17.53
	6R-3B	NRR, PF	5.36	12.83	0.67	13.77
	8R-1B	NRR, PF	2.89	12.40	0.49	14.51
	8R-2A	NRR	3.63	16.99	0.52	27.40
	8R-2B	NRR	3.16	15.61	0.24	32.54
	8R-3A	NRR, PF	2.79	13.33	0.52	16.77
	8R-3B	NRR, PF	4.27	11.72	0.45	24.82
4	9TF-3A	NRR, PF	2.96	12.74	0.17	0.76
	9TF-3B	NRR, AF	4.23	23.07	0.25	3.16
	9TF-4A	NRR, AF	2.65	21.42	0.20	2.69
	12TF-3A	NRR, AF	2.68	17.47	0.26	50.89
	12TF-3B	NRR, AF	4.01	18.74	0.12	41.70
	12TF-4B	NRR, AF	4.18	23.27	-	57.40
5	9TF-2A	NRR, AF	1.73	11.20	0.31	18.61
	9TF-2B	RRE, PF	1.10	7.44	0.19	2.10
	12TF-1B	NRR	1.76	9.90	0.22	4.58
	12TF-7A	NRR	1.23	10.33	0.17	3.18
	12TF-7B	NRR, AF	1.26	7.40	0.12	21.76
6	9R-1A	NRR	3.53	9.72	0.08	0.83
	9R-2A	RRE, PF	4.25	8.84	0.21	1.13
	9R-2B	RRE, PF	3.33	9.85	0.42	1.21
	9R-3A	RRE, SCL	3.74	14.08	-	21.39
	9R-3B	RRE, PF	3.14	14.08	0.18	7.97
	12R-1A	NRR, AF	3.26	9.41	0.20	2.21
	12R-2A	NRR, AF	4.21	14.63	0.03	16.72
	12R-2B	NRR	3.37	14.93	0.44	32.83

* NRR = No relative rotation, RRE = Relative rotation just at the end, SRR = Slight relative rotation, PF = Progressive failure, AF = Abrupt failure, SCL = Spalling under concentrated load.

**Table 4:** Mechanical parameters.

		$\tau_{1st-crack}/\tau_{max}\;(\%)$		$\tau_{max}\;(\mathrm{MPa})$		$w_{peak}\;(\mathrm{mm})$		$w_{ch}/w_{peak}\;(\%)$		$G_{fII}\;(N/\mathrm{mm})$	
Group	N° Prisms	Avg.	CV	Avg.	CV	Avg.	CV	Avg.	CV	Avg.	CV
W	11	47.61	0.22	14.39	0.37	0.67	0.62	82.42	0.37	23.53	12.87
R	18	35.22	0.28	15.01	0.18	0.36	0.49	95.95	0.48	18.19	11.42
TF-T	6	39.60	0.33	23.65	0.20	0.20	0.26	107.35	0.50	32.80	30.94
TF-L	5	63.69	0.08	11.23	0.17	0.20	0.32	111.56	0.57	12.19	10.17

## Data Availability

The data presented in this study are available on request from the corresponding author.

## References

[1] Abdelrazik A.T., Khayat K.H. (2020). Effect of Fiber Characteristics on Fresh Properties of Fiber-Reinforced Concrete with Adapted Rheology. Constr. Build. Mater..

[2] Wang W., Shen A., Lyu Z., He Z., Nguyen K.T.Q. (2021). Fresh and Rheological Characteristics of Fiber Reinforced Concrete—A Review. Constr. Build. Mater..

[3] de Figueiredo A.D., Ceccato M.R. (2015). Workability Analysis of Steel Fiber Reinforced Concrete Using Slump and Ve-Be Test. Mater. Res..

[4] Wang L., Guo F., Yang H., Wang Y., Tang S. (2021). Comparison of Fly Ash, Pva Fiber, Mgo and Shrinkage-Reducing Admixture on the Frost Resistance of Face Slab Concrete Via Pore Structural and Fractal Analysis. Fractals.

[5] Wang L., He T., Zhou Y., Tang S., Tan J., Liu Z., Su J. (2021). The Influence of Fiber Type and Length on the Cracking Resistance, Durability and Pore Structure of Face Slab Concrete. Constr. Build. Mater..

[6] Afroughsabet V., Biolzi L., Ozbakkaloglu T. (2016). High-Performance Fiber-Reinforced Concrete: A Review. J. Mater. Sci..

[7] Akeed M.H., Qaidi S., Ahmed H.U., Faraj R.H., Majeed S.S., Mohammed A.S., Emad W., Tayeh B.A., Azevedo A.R.G. (2022). Ultra-High-Performance Fiber-Reinforced Concrete. Part V: Mixture Design, Preparation, Mixing, Casting, and Curing. Case Stud. Constr. Mater..

[8] Meda A., Minelli F., Plizzari G.A. (2012). Flexural Behaviour of RC Beams in Fibre Reinforced Concrete. Compos. B Eng..

[9] Conforti A., Minelli F., Tinini A., Plizzari G.A. (2015). Influence of Polypropylene Fibre Reinforcement and Width-to-Effective Depth Ratio in Wide-Shallow Beams. Eng. Struct..

[10] Vandewalle L. (2000). Cracking Behaviour of Concrete Beams Reinforced with a Combination of Ordinary Reinforcement and Steel Fibers. Mater. Struct..

[11] Liu X., Pareek T., Chao S.-H. (2016). New Methodology for Design and Construction of Concrete Members with Complex Stress Fields Using Steel Fiber–Reinforced Concrete. J. Struct. Eng..

[12] Campione G. (2012). Flexural Behavior of Steel Fibrous Reinforced Concrete Deep Beams. J. Struct. Eng..

[13] Yousef A.M., Tahwia A.M., Marami N.A. (2018). Minimum Shear Reinforcement for Ultra-High Performance Fiber Reinforced Concrete Deep Beams. Constr. Build. Mater..

[14] Pujadas P., Blanco A., De La Fuente A., Aguado A. (2012). Cracking Behavior of FRC Slabs with Traditional Reinforcement. Mater. Struct./Mater. Et Constr..

[15] Michels J., Waldmann D., Maas S., Zürbes A. (2012). Steel Fibers as Only Reinforcement for Flat Slab Construction—Experimental Investigation and Design. Constr. Build. Mater..

[16] Facconi L., Minelli F., Plizzari G. (2016). Steel Fiber Reinforced Self-Compacting Concrete Thin Slabs—Experimental Study and Verification against Model Code 2010 Provisions. Eng. Struct..

[17] Campione G., Papia M., Fossetti M. (2010). Behavior of Fiber-Reinforced Concrete Columns under Axially and Eccentrically Compressive Loads. ACI Struct. J..

[18] Conforti A., Tiberti G., Plizzari G.A., Caratelli A., Meda A. (2017). Precast Tunnel Segments Reinforced by Macro-Synthetic Fibers. Tunn. Undergr. Space Technol..

[19] Abbas S., Soliman A.M., Nehdi M.L. (2014). Mechanical Performance of RC and SFRC Precast Tunnel Lining Segments: A Case Study. ACI Mater. J..

[20] Minelli F., Plizzari G.A. (2013). On the Effectiveness of Steel Fibers as Shear Reinforcement. ACI Struct. J..

[21] Matos L.M.P., Barros J.A.O., Ventura-Gouveia A., Calçada R.A.B. (2020). Constitutive Model for Fibre Reinforced Concrete by Coupling the Fibre and Aggregate Interlock Resisting Mechanisms. Cem. Concr. Compos..

[22] Walraven J.C. (1981). Fundamental Analysis of Aggregate Interlock. J. Struct. Div..

[23] Kaufmann W., Amin A., Beck A., Lee M. (2019). Shear Transfer across Cracks in Steel Fibre Reinforced Concrete. Eng. Struct..

[24] Abrishambaf A., Cunha V.M.C.F., Barros J.A.O. (2014). The Influence of Fibre Orientation on the Post-Cracking Tensile Behaviour of Steel Fibre Reinforced Self-Compacting Concrete. Frat. Ed Integrità Strutt..

[25] Barros J.A.O., Foster S.J. (2018). An Integrated Approach for Predicting the Shear Capacity of Fibre Reinforced Concrete Beams. Eng. Struct..

[26] Khanlou A., MacRae G.A., Scott A.N., Hicks S.J., Clifton G.C. Shear Performance of Steel Fibre Reinforced Concrete. Proceedings of the Australasian Structural Engineering Conference 2012: The Past, Present and Future of Structural Engineering.

[27] Boulekbache B., Hamrat M., Chemrouk M., Amziane S. (2012). Influence of Yield Stress and Compressive Strength on Direct Shear Behaviour of Steel Fibre-Reinforced Concrete. Constr. Build. Mater..

[28] Mirsayah A.A., Banthia N. (2002). Shear Strength of Steel Fiber-Reinforced Concrete. ACI Mater. J..

[29] Khaloo A.R., Kim N. (1997). Influence of Concrete and Fiber Characteristics on Behavior of Steel Fiber Reinforced Concrete under Direct Shear. ACI Mater. J..

[30] Soetens T., Matthys S. (2017). Shear-Stress Transfer across a Crack in Steel Fibre-Reinforced Concrete. Cem. Concr. Compos..

[31] Li B., Maekawa K. (1987). Contact Density Model for Cracks in Concrete. IABSE Colloquium.

[32] Bažant Z.P., Gambarova P. (1980). Rough Cracks in Reinforced Concrete. J. Struct. Div..

[33] Pfyl T. (2003). Tragverhalten von Stahlfaserbeton.

[34] Htut T.N.S., Foster S.J., Oh B.H., Choi K., Hong S. (2010). Unified Model for Mixed Mode Fracture of Steel Fibre Reinforced Concrete. Fracture Mechanics of Concrete and Concrete Structures—High Performance, Fiber Reinforced Concrete, Special Loadings and Structural Applications.

[35] Lee S.-C., Cho J.-Y., Vecchio F.J. (2013). Simplified Diverse Embedment Model for Steel Fiber- Reinforced Concrete Elements in Tension. ACI Mater. J..

[36] Picazo A., Alberti M.G., Gálvez J.C., Enfedaque A. (2021). Shear Slip Post-Cracking Behaviour of Polyolefin and Steel Fibre Reinforced Concrete. Constr. Build. Mater..

[37] (2010). Test Method for Shear Strength of Steel Fiber Reinforced Concrete.

[38] Fédération Internationale de la Précontrainte (1978). Shear at the Interface of Precast and in Situ Concrete: FIP Technical Report.

[39] Walraven J.C., Vos E., Reinhardt H.W. (1979). Experiments on Shear Transfer in Cracks in Concrete. Part I: Description of Results.

[40] Walraven J.C. (1979). Experiments on Shear Transfer in Cracks in Concrete. Part II: Analysis of Results.

[41] de Lima Araújo D., Lobo F.A., Martins B.G. (2021). A Shear Stress-Slip Relationship in Steel Fibre-Reinforced Concrete Obtained from Push-off Testing. Constr. Build. Mater..

[42] Maekawa K., Okamura H., Pimanmas A. (2003). Non-Linear Mechanics of Reinforced Concrete.

[43] (2005). Test Method for Metallic Fibered Concrete—Measuring the Flexural Tensile Strength (Limit of Proportionality (LOP), Residual).

[44] Mendes de Andrade R.G., Pfeil M.S., Battista R.C., Toledo Filho R.D., Oliveira de Araújo O.M., Lopes R.T. (2021). Comparison between Methods to Determine the Fibre Orientation Factor of an HPFRC Bridge Box Girder. Constr. Build. Mater..

[45] Departamento Nacional de Infraestrutura de Transportes (1996). Manual de Projeto de Obras-de-Arte Especiais, Design Manual.

[46] (2013). Road and Pedestrian Live Load on Bridges, Viaducts, Footbridges and Other Structures.

[47] S. Activity Group, fib (2011). Model Code 2010, Vols 1 and 2, Final Draft.

[48] Marangon E. (2011). Caracterização Material e Estrutural de Concretos Autoadensáveis Reforçados com Fibras de Aço.

[49] Cuenca E., Conforti A., Monfardini L., Minelli F. (2020). Shear Transfer across a Crack in Ordinary and Alkali Activated Concrete Reinforced by Different Fibre Types. Mater. Struct..

[50] AFGC (2013). Ultra High Performance Fibre-Reinforced Concretes. Recommendations.

[51] Krenchel H. Fibre Spacing and Specific Fibre Surface. Proceedings of the RILEM Symposium on Fibre Reinforced Cement and Concrete.

[52] Thrane L.N. (2013). Guideline for Execution of Steel Fibre Reinforced SCC.

[53] Bentur A., Mindess S. (2006). Fibre Reinforced Cementitious Composites.

[54] Sun Q., Martin B., Williams B., Heard W., Frew D., Nie X. (2022). Comparative Study on the Impact-Induced Microstructural Damage in Concrete Using X-Ray Computed Micro-Tomography. Mech. Mater..

[55] Ferreira S.R., de Andrade R.G.M., de Andrade G.M., de Araújo O.M.O., Lopes R.T., Fairbairn E.d.M.R., Grabois T.M., Ukrainczyk N. (2022). Bond Behavior of a Bio-Aggregate Embedded in Cement-Based Matrix. Materials.

[56] Lorenzoni R., Tinoco M., Paciornik S., de Andrade Silva F. (2021). The Use of X-Ray Microtomography to Investigate the Shear Behavior of Hybrid Fiber Reinforced Strain Hardening Cementitious Composites. J. Build. Eng..

[57] Bastos L.F., de Araújo O.M.O., Machado A.S., Oliveira D.F., Lopes R.T. (2020). X-Ray Microtomography System Applied in Characterization of Lightweight Concrete Structures. J. Nondestruct. Eval. Diagn. Progn. Eng. Syst..

[58] Abràmoff M.D., Magalhães P.J., Ram S.J. (2004). Image Processing with ImageJ. Biophotonics Int..

[59] Soroushian P., Lee C.-D. (1990). Distribution and Orientation of Fibers in Steel Fiber Reinforced Concrete. ACI Mater. J..

[60] Dupont D., Vandewalle L. (2005). Distribution of Steel Fibres in Rectangular Sections. Cem. Concr. Compos..

[61] Appa Rao G., Sreenivasa Rao A. (2009). Toughness Indices of Steel Fiber Reinforced Concrete under Mode II Loading. Mater. Struct..

[62] Soltanzadeh F., Barros J.A.O., Santos R.F.C. (2015). High Performance Fiber Reinforced Concrete for the Shear Reinforcement: Experimental and Numerical Research. Constr. Build. Mater..

